# Inflammation-related genes up-regulated in schizophrenia brains

**DOI:** 10.1186/1471-244X-7-46

**Published:** 2007-09-06

**Authors:** Peter Saetre, Lina Emilsson, Elin Axelsson, Johan Kreuger, Eva Lindholm, Elena Jazin

**Affiliations:** 1 Department of Development and Genetics, Uppsala University, Sweden; 2 Department of Genetics and Pathology, Uppsala University, Rudbeck Laboratory, Sweden

## Abstract

**Background:**

Multiple studies have shown that brain gene expression is disturbed in subjects suffering from schizophrenia. However, disentangling disease effects from alterations caused by medication is a challenging task. The main goal of this study is to find transcriptional alterations in schizophrenia that are independent of neuroleptic treatment.

**Methods:**

We compared the transcriptional profiles in brain autopsy samples from 55 control individuals with that from 55 schizophrenic subjects, subdivided according to the type of antipsychotic medication received.

**Results:**

Using global and high-resolution mRNA quantification techniques, we show that genes involved in immune response (GO:0006955) are up regulated in all groups of patients, including those not treated at the time of death. In particular, IFITM2, IFITM3, SERPINA3, and GBP1 showed increased mRNA levels in schizophrenia (p-values from qPCR ≤ 0.01). These four genes were co-expressed in both schizophrenic subjects and controls. In-vitro experiments suggest that these genes are expressed in both oligodendrocyte and endothelial cells, where transcription is inducible by the inflammatory cytokines TNF-α, IFN-α and IFN-γ.

**Conclusion:**

Although the modified genes are not classical indicators of chronic or acute inflammation, our results indicate alterations of inflammation-related pathways in schizophrenia. In addition, the observation in oligodendrocyte cells suggests that alterations in inflammatory-related genes may have consequences for myelination. Our findings encourage future research to explore whether anti-inflammatory agents can be used in combination with traditional antipsychotics for a more efficient treatment of schizophrenia.

## Background

Genome-wide expression analysis can be used to identify associations between gene products and disease independently of a-priori hypothesis. This approach has been used several times to study schizophrenia post-mortem brain tissues as reviewed previously [1,2]. Even though these studies included a very limited number of brain samples, multiple replications support a decreased expression of oligodendrocyte and myelin-related genes in schizophrenia [3-7]. However, transcriptional disturbances in patients are by nature subject to the confounding effect of the medication used to treat the disease, and this issue has received little attention, partly due to sample size limitations for studies based on human autopsy brain samples.

We have previously used a hypothesis driven approach to examine the expression levels of the RNA binding protein QKI and six oligodendrocyte related (OR) genes in post-mortem frontal cortex samples [8,9]. Our results indicated that QKI regulates the mRNA levels of myelin and oligodendrocyte genes in the human brain, and that changes in the balance between QKI splice variants may be linked to altered myelination in schizophrenia. We also showed, using a large collection of brain autopsies from 55 patients and 55 controls, that the modification in OR gene expression varied with the type of medication received [8]. These results demonstrated the importance of incorporating medication as a factor in the analysis of transcriptional changes in schizophrenia.

In this study, we have widened our scope and use a data driven approach to identify additional transcriptional changes that are independent of the medication received by the patients. There was only one gene ontology (GO) category that was over-represented among genes with modified expression in schizophrenic subjects that was independent of medication, and four of the genes in this group are induced by inflammatory cytokines. Our results may provide initial molecular markers to support the perhaps controversial theory that postulates that inflammation or inflammation-related processes may be key features behind schizophrenia [10-14]. To evaluate whether the observed transcriptional disturbance may have consequences for the micro-vascular system and myelin production in the brains of patients, we also studied the mRNA levels *in-vitro *in both endothelial and oligodendrocyte cell lines, before and after treatment with inflammatory cytokines.

## Methods

### Brain autopsies

Autopsies from 110 individuals were obtained from three brain banks and each collection contained an equal number of tissue samples from individuals diagnosed with schizophrenia and individuals without psychiatric diagnosis that were used as controls. The same sample set was previously used for the analysis of myelination-related genes [8,15]. The Stanley Foundation Brain Consortium (Bethesda, USA) contributed with brain autopsies from 30 individuals, 48 samples were provided by Maudsley Brain Bank (Institute of Psychiatry, Dept. of Neuropathology, London, UK) and 32 came from Harvard Brain Tissue Resource Centre (Massachusetts General Hospital, Massachusetts, USA). The diagnostic criterion used by the Stanley foundation was DSM-IV, whereas the Maudsley brain bank used ICD-10 and the Harvard Resource Centre used the Feighner criteria. Forty-six samples were from females and 64 were from males. In total 110 tissue samples from frontal cortex, Brodmann area 8 and 9 (from Harvard and Stanley brain banks) and the left side of the superior frontal gyrus (from Maudsley brain bank), were included in the study. The mean time post mortem was 34.3 hours for controls (standard deviation 22.6) and 34.6 hours for schizophrenic subjects (standard deviation 20.5), where as the average age of controls and affected subjects were 58.5 (18.0) and 54.7 (17.3), respectively. Fifty-five tissue samples came from controls (CTRL) and 55 from patients. Seven of the schizophrenic (SCH) individuals were treated with atypical neuroleptics (ATYP), nineteen were treated with typical neuroleptics (TYP) and eleven schizophrenic individuals were not treated with neuroleptics prior to death (NTD). For the remaining eighteen schizophrenic individuals no information regarding medication was available (UNKNW). The typical neuroleptics group included patients treated with chlorpromazine, promazine, haloperidol, thioridazine, stelazine, trifluroperazine, thiothixene and sulpiride. The atypical neuroleptics group included patients treated with clozapine and risperidone. Each autopsy sample consisted of 300 mg to 500 mg of prefrontal cortex tissue with similar amounts of grey and white matter in each sample. Moreover, duplicate autopsies were collected from each individual to control for differences in the proportion of white matter in each sample.

Demographic information about each individual has been loaded to the miame express database (ArrayExpress accession number E-MEXP-857) and a detailed description of each subject can also be found in Supplementary Table 1. Ethical approval for the use of these samples was received according to Swedish regulations (Ups dnr 99277).

### Microarray hybridization

To identify genes that differed in mRNA expression levels between all schizophrenic patients and controls or between a patient subgroup and controls we carried out several different microarray experiments. Messenger RNA from all individuals was combined into five sample pools, with each individual in a pool contributing with an equal amount of mRNA. The mRNA samples were pooled as follows: patients treated with typical neuroleptics (TYP, n = 19), patients treated with atypical neuroleptics (ATYP, n = 7), patients that did not receive any neuroleptics at the time of death (NTD, n = 11), all control individuals (CTRL, n = 55), and all schizophrenic patients (n = 55). One hundred ng from each of the four schizophrenic mRNA pool was hybridized together with 100 ng mRNA from the control pool on slides printed with 29 750 human cDNA clones [16]. Detailed information on the array designs is available on ArrayExpress (accession number A-MEXP-114). Each hybridization was repeated four times, with the dye assignment reversed in the two of the hybridizations (dye swap pairs), resulting in a total of 16 microarrays.

The MICROMAX TSA™ Labeling and Detection kit (NEN^® ^Life Science Products, Inc.) was used to label the sample pools with Cyanine-3 (Cy-3) and Cyanine-5 (Cy-5) respectively. The TSA™ procedure was performed according to manufacturer's protocols.

### Microarray analysis

The microarrays were scanned at 10 μm resolution using a GenePix 4100A scanner (Axon Instruments, Inc.). Spots on the resulting images were quantified with the software package GenePix Pro 5.0 (Axon Instruments, Inc.). The mean intensity of the two samples (Cy5-labelled sample = R, the Cy3-labelled sampled = G) were used to calculate the log-transformed ratio between the two samples for each spot: M = log_2 _(R/G). Microarray data was submitted to ArrayExpress.

Prior to the analysis of the microarray data, we used a robust scatter plot smoother (Proc Loess, SAS v8.2) to perform a sub-array intensity-dependent normalization of M, with the smoothing parameter set to 40%. All spots with a mean spot intensity below the local median background were excluded from the analysis. To identify genes that were differentially expressed in at least one patient subgroup, we analyzed the signals from the clones on all 16 arrays with a regression model (Proc GLM, SAS v8.2). In the model the normalized log-transformed ratio y of array j was modelled as a function of schizophrenic subgroup:

yj=β0+∑i=14βixi+ε
 MathType@MTEF@5@5@+=feaafiart1ev1aaatCvAUfKttLearuWrP9MDH5MBPbIqV92AaeXatLxBI9gBaebbnrfifHhDYfgasaacH8akY=wiFfYdH8Gipec8Eeeu0xXdbba9frFj0=OqFfea0dXdd9vqai=hGuQ8kuc9pgc9s8qqaq=dirpe0xb9q8qiLsFr0=vr0=vr0dc8meaabaqaciaacaGaaeqabaqabeGadaaakeaacqWG5bqEdaWgaaWcbaGaemOAaOgabeaakiabg2da9GGaciab=j7aInaaBaaaleaacqaIWaamaeqaaOGaey4kaSYaaabCaeaacqWFYoGydaWgaaWcbaGaemyAaKgabeaaaeaacqWGPbqAcqGH9aqpcqaIXaqmaeaacqaI0aana0GaeyyeIuoakiabdIha4naaBaaaleaacqWGPbqAaeqaaOGaey4kaSIae8xTdugaaa@43A7@

where *β*_0 _referrers to the intercept, *x*_*i *_refers to the four design variables NTD, TYP, ATYP and UNKWN, and *β*_*i *_to the corresponding regression coefficient estimating the mRNA difference between subgroup *i *and the control. The arrays containing samples from all the schizophrenic patients was represented by the sum of all four design variables, each multiplied by the fraction of individual contributing to the specific subgroups. To identify common mRNA responses in all the three subgroups with known medication status, we also tested whether the mean of the three estimated responses (*β*_*i*_) differed from zero (n = 3). We penalized all F-ratio by adding a constant (a_0_) to the denominator and choose a_0 _to be the 90^th ^percentile of the mean square errors of all analyzed clones. We considered penalized F-ratios above 7 to be indicative of a differential expression for a schizophrenic subgroup, and a penalized F-ratio above 2.2 to indicate a common response in the three subgroups as determined by a 10,000 permutations. The cut-offs values corresponded to four expected false positives for each clone list.

Overrepresentation of a biological function within a list of selected genes was assessed with GOstat [17,18]. That is, to assess statistically overrepresentation we compared the observed frequencies of genes within Gene Ontology (GO) classes for a list of selected clones with the frequencies of all genes represented by all analyzed clones, using the FDR option to correcting for multiple testing. We only considered the biological function hierarchy, and used a minimal GO path length of five.

### Real-time RT PCR

All samples, without pooling, were analysed by real-time RT PCR. Tissue sample preparation, total RNA preparation, poly-A RNA purification as well as the reverse transcription reactions were performed as previously described [19]. Briefly, total RNA was prepared using Trizol reagent (Life Technologies, Sweden), mRNA was extracted using the PolyATtract mRNA isolation system (Promega SDS, Sweden) and reverse transcription employed Superscript II (Life Technologies, Sweden). The 110 individuals, covering 55 patients and 55 controls, were distributed in duplicates on three plates according to a balanced incomplete block design, with respect to diagnosis, sex and brain bank. Real-time RT-PCR was performed with an ABI PRISM 7000 Sequence Detection System (Applied Biosystems, Foster City, USA) as follows: 2 minutes at 50°C and 10 minutes at 95°C followed by 40 cycles of 15 seconds at 95°C and 1 minutes at 60°C. Each reaction was carried out in a total volume of 25 μl, consisting of 9.8 μl TaqMan universal PCR master mix, 0.125 μM probe (Applied Biosystems, Foster City, USA), 0.25 μM of each primer (Thermo Electron Cooperation, Germany) and ~ 10–100 ng of cDNA.

The primer/probe-set for the reference genes (ACTB and GAPD) as well as for the IFITM2, IFITM3 and MAG were uniquely designed using Primer Express (Applied Biosystems, Foster City, USA). Primers for TF, SCD, SERPINA3, GBP1 and HLA-A were ordered as "Assay on Demand" (Applied Biosystems, Foster City, USA). The expression data was collected with the ABI PRISM 7000 SDS software (Applied Biosystems, Foster City, USA).

### qPCR analysis

To test if the expression of the target genes were disturbed in patients suffering from schizophrenia and/or affected by patient sub-group with respect to neuroleptic treatment, we analyzed the mRNA levels with an ANCOVA model (Proc GLM, SAS 8.2) as we previously described [8]. The model included the main factors *disease *(SCH/CTRL), and *disease subgroup *(ATYP, TYP, NTD) nested within *disease *status. Thus, the 18 patients with unknown medication status were included in the comparisons of all patients versus controls, but they were excluded in the test of patient subgroups. In addition, we included the categorical factors brain bank, gender, and replicate PCR-plate, and the covariates, age, post-mortem time and reference gene expression (the geometric mean of ACTB and GAPD) to account for the effects of these variables on mRNA levels of the target genes. We used logarithmic transformed expression data and averaged the mRNA levels obtained from the duplicate samples from each individual prior to the statistical analysis.

The co-expression patterns of genes that were differentially expressed in schizophrenic subjects and/or varied between schizophrenic subgroups (IFITM2, IFITM3, SERPINA3, GBP1, SCD, MAG and TF) were explored for schizophrenic subjects and controls respectively. Target gene expression was normalized, prior to the analysis, by fitting the linear model described above to the observed data (Proc GLM, SAS 8.2). The difference between the observed and the fitted values (i.e. the residuals) were considered the normalized expression level, and all pair-wise Pearson correlation coefficients were calculated.

### Cell cultures and interferon treatment

Human telomerase-immortalized dermal microvascular endothelial cells (TIME) [20], were cultured in EBM MV2 growth medium (Promocell). The human oligodendrocyte-derived cell line (HOG)[21] was kept in Dulbecco's modified Eagle's medium (DMEM) with low glucose and GlutaMAX (Invitrogen), supplemented with 5% fetal bovine serum and 1% penicillin/streptavidin. TIME and HOG cells were treated for 3, 8, 24 or 48 hours with either 10 ng/ml IFN-γ (Peprotech), 10 ng/ml IFN-α (Peprotech) or 1 ng/ml TNF-α (R&D), and thereafter harvested for further analysis. All cell culture experiments were performed as independent triplicates. To test if the expression levels of inflammatory genes were induced by cytokines in human cell lines, we analyzed the mRNA levels of target genes in each experiment with an ANCOVA model (Proc GLM, SAS 8.2). The model included the main factors, treatment (Cytokine addition vs CTRL), and time (3, 8, 24, 48 h) nested within treatment. In addition, we included reference gene expression (ACTB) to account for the systematic variation that was due to sample mRNA concentration. Expression levels of target and reference genes were log-transformed prior to the statistical analysis.

## Results

### Analysis of expression differences that are independent of medication received

We first identified genes with a high probability of being differentially expressed independently of neuroleptic medication. Twenty-three clones showed large and consistent mRNA differences across all three subgroups, as indicated by the penalized F-ratio (Table 1). This number of clones is approximately six times higher than the expected number of four false positives (as determined by 10 000 permutations). When these 23 genes were classified with respect to their biological function, genes involved in immune response (GO:0006955) corresponded to approximately 25% of the GO-annotated genes. This represents a strong overrepresentation, since the frequency of immune response genes was only 2.6% of all analyzed and annotated clones on the array (*p*-value < 0.01). The five genes involved in immune response, alpha-1-antichymotrypsin (SERPINA3), interferon-induced transmembrane 2 and 3 (IFITM2 and IFITM3), guanylate binding protein 1, interferon inducible (GBP1) and major histocompatibility complex, class1, A (HLA-A), were all up-regulated in schizophrenic patients (marked with "*" in Table 1), indicating disturbances in inflammatory-related responses.

**Table 1:** Clones with differential expression patterns across all patient subgroups

		Fold Change					
					
Clone	Symbol	NTD (n = 11)	Typical (n = 19)	Atypical (n = 7)	Penalized F-ratio	*p*-value	Location
1592837	IFITM2*	2.7	1.5	2.1	11.3	0.024	11p15.5
809910	IFITM3*	4.3	1.6	1.8	10.1	0.032	11p15.5
450533	SERPINA3*	4.5	1.2	7.8	8.4	0.056	14q32.1
1455976	IFITM2*	2.3	1.4	2.3	7.0	0.061	11p15.5
166245	GRIN1	-1.6	-1.3	-1.4	4.7	0.004	9q34.3
362926	PRKACB	1.4	1.7	1.5	4.6	0.012	1p36.1
323371	APP	-2.9	-1.8	-1.7	4.2	0.083	21q21.2
841008	GBP1*	2.0	1.7	-1.0	4.1	0.079	1p22.2
221828	KIF1A	-1.3	-1.5	-1.5	4.0	0.005	2q37.3
853906	HLA-A*	1.1	1.5	1.6	3.8	0.045	6p21.3
363007	AGXT2L1	-1.2	-1.3	-2.5	3.7	0.073	4q25
756556	SERPING1	1.3	1.2	1.4	3.6	0.048	11q12-q13.1
214162	MT1H	1.5	1.5	1.1	3.5	0.056	16q13
41447	AGTRL1	1.3	1.2	1.3	3.2	0.025	11q12
45544	TAGLN2	1.3	1.4	1.1	3.1	0.082	1q21-q25
44310	ELL	-1.9	1.3	-3.1	2.9	0.153	19p13.1
487777	RB1	1.1	1.3	1.4	2.8	0.063	13q14.2
245774	C10orf10	1.4	-1.1	2.2	2.8	0.136	10q11.21
173145	CLIPR-59	-2.1	-1.1	-1.2	2.8	0.091	19q13.12
703479	HDHD1A	1.2	1.3	1.3	2.7	0.010	Xp22.32
244147	*In multiple UG-clusters*	1.3	2.2	1.2	2.7	0.095	
839374	EXTL2	-1.6	-1.3	-1.1	2.7	0.032	1p21
1908973	CART	1.1	1.3	1.4	2.6	0.052	5q13.2

Schizophrenic patients were subdivided according to the antipsychotic medication received at death, and pooled brain samples were hybridized onto human cDNA microarrays. Twenty-three clones with large and consistent mRNA differences in all three sub-groups are listed together with their ranking statistic and parametric p-value. Four false positives are expected by chance in the list. Genes with a biological function involved in immune response (*, GO:0006955) were overrepresented in the list (*p*-value < 0.01). Listed *P*-values correspond to the parametric test of the average effect (across the subgroups) being different from the control (n = 3).

### Analysis of expression differences for each of the three patient subgroups separately

**Figure 1:**
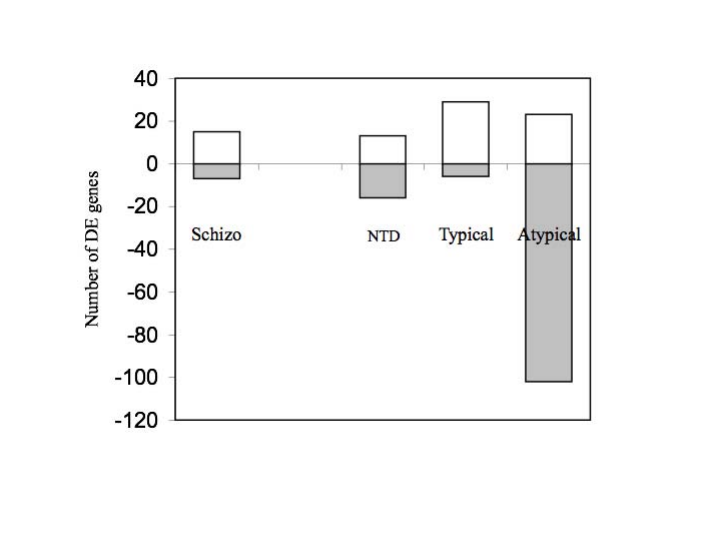
**Number of clones showing evidence of differential expression in at least one subgroup of schizophrenic patients**. Schizophrenic patients were subdivided according to the type of antipsychotic medication received at death, and pooled brain samples were hybridized onto 29.8 K cDNA microarrays. Bars represent the number of clones showing evidence of increased (white) or decreased (grey) mRNA levels in all (Schizo) or in at least in one of three schizophrenic subgroups (NTD, Typical, Atypical), as compared to control individuals. Detailed lists of clones for each subgroup used to construct the figure are included as Supplementary Tables 2, 3, and 4. The genes that were differentially expressed independently of received medication (Schizo) are listed in Table 1.

After the screening for genes that were differentially expressed in schizophrenia independently of received neuroleptic treatment, we identified clones that showed a large average expression difference between schizophrenic patients and controls, as compared to random technical variation, for *each of the three patient subgroups separately*. (The cut-off value for the ranking statistics was adjusted so that four false positives were expected in each list due to random technical variation). We note that this analysis served a primarily exploratory purpose, as pooling does not allow for estimation of population variance. Consequently the results from any individual genes (Supplementary tables 2–5) should be interpreted with extreme caution, until mRNA levels have been quantified on an individual level. Nevertheless, results based on multiple genes are less likely to be affected by a few extreme values and our results suggest that both the number and direction of differentially expressed genes may vary between the three subgroups (Figure 1). In particular we note that the group treated with atypical neuroleptics had approximately four times as many genes showing an average expression deviations from the control group than other schizophrenic subjects, and that the distribution of clones showing increased or decreased mRNA levels strongly depended on schizophrenic subgroups (p-value < 0.0001, Fishers exact test). Although these preliminary findings need to be confirmed on an individual level similar trends have been reported from experiments with haloperidol and risperidone on rats [22].

### Real-time RT-PCR

Since the micro array screening for genes with common response across all schizophrenic subgroups suggested that genes involved in immune response (GO:0006955) were of particular interest, we quantified mRNA levels of five genes related to immune response (Table 1) in all samples without pooling, using real-time reverse transcription PCR (qPCR). These genes included SERPINA3, IFITM2, IFITM3, HLA-A, and GBP1 (Table 2). We compared the results from these genes with qPCR resuts obtained for oligodendrocyte-related genes, including transferrin (TF), Myelin associated glycoprotein (MAG) and Stearoyl-CoA desaturase (SCD), as disturbed expression of oligodendrocyte releated genes have been repeatedly reported in schizophrenia, and as all three genes were clearly down regulated on the arrays from subjects treated with atypical neuroleptics (Supplementary table 5). Overall, there was a good correspondence between mRNA levels estimated in the three patient subgroups by microarray and qPCR analysis and the qPCR results confirmed the microarray findings with the exception of HLA-A (r^2 ^= 0.71, n = 21). That is, SERPINA3, IFITM2, IFITM3, and GBP1 showed significantly higher qPCR mRNA levels in schizophrenic subjects than in control individuals and this response was shared by all three patient subgroups (Table 2).

**Table 2:** Messenger RNA levels of immune response and oligodendrocyte related genes in frontal cortex of schizophrenic subjects as compared to control individuals, quantified by qPCR

	Fold change				F-ratio			
		
Gene	Schizo (n = 55)	NTD (n = 11)	Typical (n = 7)	Atypical (n = 7)	Schizo vs. Ctrl		Subgroup	
SERPINA3	3.6	4.4	2.2	7.6	20.7	***	1.8	
IFITM2	1.4	1.7	1.3	1.6	16.2	***	1.8	
IFITM3	1.4	1.7	1.2	1.5	16.3	***	2.7	
GBP1	1.5	1.6	1.6	2.0	6.9	**	0.1	
HLA-A	1.2	1.0	1.3	1.2	2.6		1.0	
MAG	-1.7	-1.8	-1.4	-3.5	9.5	**	2.2	
SCD	-1.2	-1.5	1.1	-2.1	3.3		6.9	**
TF	-1.4	-1.9	1.1	-3.2	5.1	*	7.9	***

Schizophrenic patients have been subdivided according to the antipsychotic medication received at death. Mean levels across all schizophrenic subjects are compared to controls individuals (Schizo vs. Ctrl), and the heterogeneity between the three subgroups is tested (Subgroup). Asterisk (*) indicates statistically significant deviation from the null hypothesis (p-value <0.05, <0.01 and <0.001 for one, two and three asterisks, respectively).

To rule out the possibility that increased mRNA levels of immune response genes in schizophrenic subjects were due to infectious disease at the time of death, we performed two additional analyses. First, a senior physician classified the death cause of all 110 subjects into two categories, "likely infectious" (bronchopneumonia, intra-abdominal sepsis, multiple organ failure, peritonitis, pneumonia, pulmonary tuberculosis) or "non-likely infectious". The data was then re-analyzed, taking account of the effect of infectious/non-infectious death cause as a factor in the statistical analysis. In a second analysis we excluded all subjects that died from a "likely infectious" cause and we re-analysed the remaining patients and controls. Both these analyses confirmed that mRNA levels of SERPINA3, IFITM2, IFITM3 and GBP1 were increased in schizophrenia and were not simply the consequence of an infection immediately prior to death. *P*-values for the four genes were 0.003, 0.004, 0.004, 0.023 for the analysis accounting for infectious/non-infectious death cause, and 0.0006, 0.004, 0.003, 0.035 for the analysis excluding subjects that died due to infectious causes.

A correlation analysis indicated that the four inflammation-related genes SERPINA3, IFITM2, IFITM3 and GBP1 were co-expressed in both schizophrenic subjects and controls (Table 3). This correlation indicates that brain samples with high mRNA levels of one gene also tended to have high mRNA levels of the other three genes. This implicates that the observed disturbance in schizophrenia is indeed reflecting a coordinated transcriptional response in several inflammation-related genes. Similarly, the mRNA levels of the three OR genes (SCD, MAG and TF) were also strongly co-expressed, which is in line with our previous results on other OR genes [8]. We also note that there is no strong correlation between inflammation-related gene expression and expression of oligodendrocyte related genes, suggesting that the two sets of genes are expressed relatively independent of each other in the investigated samples.

**Table 3:** Correlation analysis of qPCR expression levels

Gene Name	SERPINA3	IFITM2	IFITM3	GBP1	MAG	SCD	TF
SERPINA3	1.00	**0.60**	**0.66**	**0.39**	0.16	0.16	0.12
IFITM2	**0.58**	1.00	**0.91**	**0.56**	0.00	0.08	-0.04
IFITM3	**0.60**	**0.90**	1.00	**0.60**	0.11	0.16	0.02
GBP1	**0.39**	**0.51**	**0.62**	1.00	0.22	0.31	0.23
MAG	-0.05	-0.16	-0.13	-0.14	1.00	**0.88**	**0.93**
SCD	0.14	-0.04	-0.04	-0.06	**0.64**	1.00	**0.89**
TF	0.14	0.01	-0.03	-0.09	**0.76**	**0.81**	1.00

The co-expression patterns of genes that were differentially expressed in schizophrenic subjects and/or varied between schizophrenic subgroups (IFITM2, IFITM3, SERPINA3, GBP1, SCD, MAG and TF) were explored for schizophrenic subjects and controls respectively. All pair-wise Pearson correlation coefficients were calculated on pPCR expression levels that remained after removing systematic variation that was due to factor such as diagnosis, schizophrenic subgroup, brain bank, age, post mortem time and reference gene expression (see details in Methods). Correlation of mRNA levels in the frontal cortex of schizophrenic subjects (below diagonal) and control individuals (above diagonal). Pearson correlation coefficients that were significant at the 0.01 levels have been outlined in bold.

### Expression of immune-related genes in oligodendroglial and endothelial cell lines

**Figure 2:**
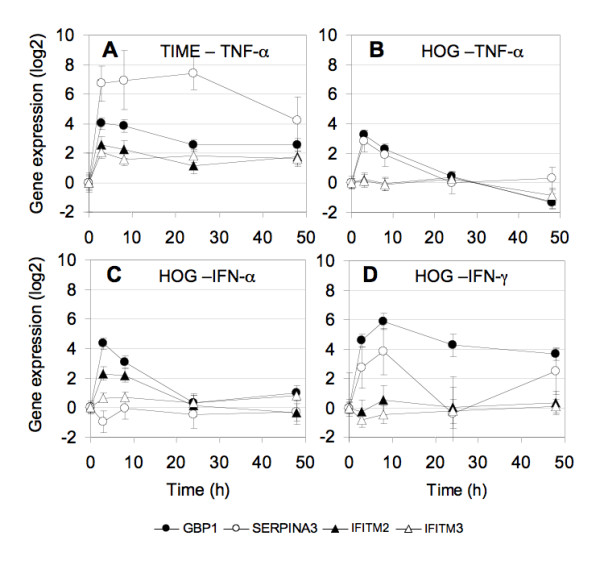
**Cytokine stimulated gene expression in human cell lines**. A human endothelial cell line (TIME) was incubated with 10 ug TNF-α during 3, 8, 24 or 48 hours (A), while human oligodendroglial (HOG) cell cultures were incubated with either10 ng/ml IFN-γ (B), 10 ng/ml IFN-α (C) or 1 ng/ml TNF-α (D). Expression levels were quantified by qPCR and are shown relative to the expression levels in non-stimulated cells (defined as "zero") on a log_2 _scale. Mean and standard error of independent triplicate cell cultures are given.

As it has been suggested that schizophrenia may be the consequence of a vascular-inflammation [14], we studied the mRNA levels of SERPINA3, IFITM2, IFITM3, and GBP1 in a human endothelial cell line (TIME), before and after stimulation with TNF-α (Figure 2A). All four genes were expressed and stimulated in the endothelial cell line. The mRNA levels of SERPINA3 and GBP1 increased 80-fold and 9.4-fold in response to TNF-α addition, and the corresponding response of IFITM2 and IFITM3 was 3.8 and 3.4-fold respectively (p-values for the four genes were 0.0215, <0.0001, 0.0200 and 0.0118).

Since the expression of myelination genes is disturbed in schizophrenia, we also decided to examine whether one or more of the inflammation-related genes were expressed in a human oligodendrocyte-cell line (HOG). Unexpectedly, all four genes were expressed in HOG cells and their expression was stimulated by inflammatory cytokines (Figure 2B, C, D). In particular, the mRNA levels of GBP1 increased 24-fold (p < 0.001) with IFN-γ stimulation. GBP1 expression was also stimulated by IFN-α and TNF-α (p-values were, <0.0001, and 0.0022), but the response was less pronounced in magnitude and time. IFITM2 expression was only induced by IFN-α (p = 0.0069) and SERPINA3 was only significantly induced by TNF-α (p = 0.05).

## Discussion

Immune-mediated inflammation is normally greatly reduced in the brain, a phenomenon called immune privilege [23]. Immune mediated inflammation can have severe consequences for the brain, which have limited capacity for regeneration. We here show that four genes involved in inflammatory response are up-regulated in post-mortem frontal cortex samples from schizophrenic subjects irrespective of neuroleptic treatment. That inflammatory factors may be involved in the disease has been scientifically discussed since the "epidemic" appearance of psychotic disorders in 1845 [24]. For example, signs of inflammation and microglia activation in post-mortem brains, a dysfunctional blood brain barrier, enhanced cytokine levels in the CSF preceding psychotic episodes, and signs of T cell system activation, all point to parallels between schizophrenia and autoimmune/infectious disorders. Other indirect evidence to support that early inflammation may contribute to the development of schizophrenia include the facts that maternal infections increase the risk of the disease [25], mice exposed to cytokines in the uterus show behavioural alterations relevant for schizophrenia [26-28], and an increased incidence of the disease during the winter months [29].

Our results give additional support to the inflammatory hypothesis for schizophrenia and provide four biological markers for the disease likely to be directly involved in inflammatory processes, namely GBP1, SERPINA3, IFITM2, and IFITM3. **GBP1**, or Interferon-gamma-inducible human guanylate binding protein 1, belongs to the family of dynamin-related large GTP-binding proteins. It is specifically induced by interferon gamma [30], and is a marker for monitoring cytokine induced phenotype of endothelial cells during inflammation [31]. **SERPINA3 **is a member of the serine protease inhibitor superfamily. It is required for the regulation of leukocyte proteases released during an inflammatory response [32], and its plasma levels increase more than four-fold in response to inflammation [33]. **IFITM2 and -3 **are members of a family of genes encoding interferon-induced transmembrane proteins. Interestingly, mouse ifitm1 and -3, which are expressed in primordial germ cells, also have important and specific roles for cell development [34].

We observed coordinated increases in the expression levels of these four genes in the brain of the schizophrenic subjects. It is unlikely that our results are driven by sporadic infectious diseases late in life, as schizophrenic subjects showed significantly elevated mRNA levels also when the effect of infectious death cause was accounted for in the analysis. Although alterations in these immune-related transcripts are not sufficient evidence for inflammation in schizophrenia, and these genes are not classic indicators of chronic or acute inflammation, our results indicate a significant alteration of inflammation-related pathways. Thus, modifications in inflammatory-related responses in the brain appear to be a common characteristic for the progression of schizophrenia for a large group of patients.

A recent theory postulates that schizophrenia may primarily be a consequence of vascular inflammation in the brain [14]. The theory proposes that abnormalities arise because genetically modulated inflammatory reactions damage the micro-vascular system in response to environmental agents such as infections, hypoxia, and physical trauma. To evaluate this hypothesis, we examined whether inflammatory cytokines can induce the expression of the four genes in a human endothelial cell line. Our *in-vitro *experiments demonstrated that GBP1, SERPINA3, IFITM2, and IFITM3, could indeed be induced by cytokine stimulation in endothelial cells. GBP1 is known as a key mediator of the anti-proliferative effect of inflammatory cytokines in endothelial cells [35]. The elevated mRNA levels in schizophrenic subjects may thus reflect changes in endothelial brain cells, with possible long-term consequences for the micro-vascular system during the progression of the disease.

Intriguingly, we also observed expression of inflammation related genes in oligodendroglial cells and induction by cytokines in these cells. Our results indicate that oligodendroglial cells may indeed contain receptors for several inflammatory cytokines and that the expression of several inflammation-related genes can be induced by these cytokines. It has recently been shown that inflammation can modify myelination levels on transplanted oligodendrocyte precursors [36]. Also, oligodendrocyte precursor responses are dependent on the presence of cytokines [37]. Thus, the observed increased expression of GBP1, IFITM2, IFITM3 and SERPINA3 in schizophrenic subjects may affect myelin producing cells, offering a possible link between the inflammatory and the myelin hypothesis of the disease.

**Pro-inflammatory **agents appear to have little or no effect on symptoms of schizophrenia [38,39]. On the other hand, **anti-inflammatory **agents have been used for the treatment of one patient, with improvement of negative symptoms [40]. Our results may encourage future clinical trials, aimed to determine whether anti-inflammatory agents in combination with traditional neuroleptics can be used as an effective treatment for positive and/or negative symptoms of schizophrenia.

## Conclusion

Although the modified genes identified in this study are not classical indicators of chronic or acute inflammation, our results indicate that inflammation-related pathways may be disturbed in the brain of schizophrenic subjects. In addition, the observations in cell cultures suggest that alterations in inflammatory-related genes may have consequences for both vascular tissue and myelination. Future research is needed to clarify whether cytokines have indeed triggered the observed inflammation-related response in the frontal cortex of schizophrenic subjects, what specific cell types have been affected, and what the consequences of these responses have for the development of the disease. In parallel to such resaerch we hope that our results may encourage future research exploring whether anti-inflammatory agents can be used in combination with traditional antipsychotics for a more efficient treatment of schizophrenia.

## Competing interests

The authors declare that they have no competing interest.

## Authors' contributions

PS conception and design, analysis and interpretation, drafting the manuscript. LE acquisition of data, revisions of the manuscript. EA analysis and interpretation. JK acquisition of data, revisions of the manuscript. EL acquisition of data, analysis and interpretation, revisions of the manuscript. EJ conception and design, analysis and interpretation, drafting the manuscript. All authors have approved the final version of the manuscript.

## Pre-publication history

The pre-publication history for this paper can be accessed here:



## Supplementary Material

Additional file 1 Supplementary Table 1 – Detailed description of studied subjects. Information about brain bank of origin, gender, age, time post-mortem (PMI), agonal state, age of onset, brain pH and medication for the 110 individuals included in the study. Click here for file

Additional file 2 Supplementary Table 2 – Genes differentially expressed in schizophrenic subjects not treated at death. List of 29 clones showing evidence of being differentially expressed in frontal cortex autopsy samples from schizophrenic subjects as compared to unaffected individuals. Results from experiments of hybridizing pooled mRNA samples to cDNA microarrays. Click here for file

Additional file 3 Supplementary Table 3 – Genes differentially expressed in patients treated with atypical neuroleptics compared to controls. List of 125 clones showing evidence of being differentially expressed in frontal cortex autopsy samples from schizophrenic subjects as compared to unaffected individuals. Results from experiments of hybridizing pooled mRNA samples to cDNA microarrays. Click here for file

Additional file 4 Supplementary Table 4 – Genes differentially expressed in patients treated with typical neuroleptics compared to controls. List of 35 clones showing evidence of being differentially expressed in frontal cortex autopsy samples from schizophrenic subjects as compared to unaffected individuals. Results from experiments of hybridizing pooled mRNA samples to cDNA microarrays. Click here for file

Additional file 5 Supplementary Table 5 – Gene expression of oligodendrocyte and myelination related genes in the frontal cortex of schizophrenic subjects as compared to control individuals. Eleven oligodendrocyte and myelination related genes that showed evidence of being differentially expressed in schizophrenic subjects in at least one medication subgroup are listed. Results from experiments of hybridizing pooled mRNA samples to cDNA microarrays. Click here for file

## References

[B1] McInnes LA, Lauriat TL (2006). RNA metabolism and dysmyelination in schizophrenia. Neurosci Biobehav Rev.

[B2] Mirnics K, Levitt P, Lewis DA (2006). Critical Appraisal of DNA Microarrays in Psychiatric Genomics. Biol Psychiatry.

[B3] Hakak Y, Walker JR, Li C, Wong WH, Davis KL, Buxbaum JD, Haroutunian V, Fienberg AA (2001). Genome-wide expression analysis reveals dysregulation of myelination-related genes in chronic schizophrenia. Proc Natl Acad Sci U S A.

[B4] Tkachev D, Mimmack ML, Ryan MM, Wayland M, Freeman T, Jones PB, Starkey M, Webster MJ, Yolken RH, Bahn S (2003). Oligodendrocyte dysfunction in schizophrenia and bipolar disorder. Lancet.

[B5] Aston C, Jiang L, Sokolov BP (2004). Microarray analysis of postmortem temporal cortex from patients with schizophrenia. J Neurosci Res.

[B6] Sugai T, Kawamura M, Iritani S, Araki K, Makifuchi T, Imai C, Nakamura R, Kakita A, Takahashi H, Nawa H (2004). Prefrontal abnormality of schizophrenia revealed by DNA microarray: impact on glial and neurotrophic gene expression. Ann N Y Acad Sci.

[B7] Dracheva S, Davis KL, Chin B, Woo DA, Schmeidler J, Haroutunian V (2006). Myelin-associated mRNA and protein expression deficits in the anterior cingulate cortex and hippocampus in elderly schizophrenia patients. Neurobiol Dis.

[B8] Aberg K, Saetre P, Jareborg N, Jazin E (2006). Human QKI, a potential regulator of mRNA expression of human oligodendrocyte-related genes involved in schizophrenia. Proc Natl Acad Sci U S A.

[B9] Aberg K, Saetre P, Lindholm E, Ekholm B, Pettersson U, Adolfsson R, Jazin E (2006). Human QKI, a new candidate gene for schizophrenia involved in myelination. Am J Med Genet B Neuropsychiatr Genet.

[B10] Stevens JR (1982). Neuropathology of schizophrenia. Arch Gen Psychiatry.

[B11] Lin A, Kenis G, Bignotti S, Tura GJ, De Jong R, Bosmans E, Pioli R, Altamura C, Scharpe S, Maes M (1998). The inflammatory response system in treatment-resistant schizophrenia: increased serum interleukin-6. Schizophr Res.

[B12] Giovannoni G, Baker D (2003). Inflammatory disorders of the central nervous system. Curr Opin Neurol.

[B13] Altamura AC, Bassetti R, Bocchio L, Santini A, Mundo E (2003). Season of birth and inflammatory response system in schizophrenia. Prog Neuropsychopharmacol Biol Psychiatry.

[B14] Hanson DR, Gottesman (2005). Theories of schizophrenia: a genetic-inflammatory-vascular synthesis. BMC Med Genet.

[B15] Sundberg R, Castensson A, Jazin E (2006). Statistical modeling in case-control real-time RT-PCR assays, for identification of differentially expressed genes in schizophrenia. Biostatistics.

[B16] Microarray Resource Centre RIT http://www.biotech.kth.se/molbio/microarray/index.html.

[B17] Gostat http://gostat.wehi.edu.au/.

[B18] Beissbarth T, Speed TP (2004). GOstat: find statistically overrepresented Gene Ontologies within a group of genes. Bioinformatics.

[B19] Castensson A, Emilsson L, Preece P, Jazin EE (2000). High-resolution quantification of specific mRNA levels in human brain autopsies and biopsies. Genome Res.

[B20] Venetsanakos E, Mirza A, Fanton C, Romanov SR, Tlsty T, McMahon M (2002). Induction of tubulogenesis in telomerase-immortalized human microvascular endothelial cells by glioblastoma cells. Exp Cell Res.

[B21] de Arriba Zerpa GA, Saleh MC, Fernandez PM, Guillou F, Espinosa de los Monteros A, de Vellis J, Zakin MM, Baron B (2000). Alternative splicing prevents transferrin secretion during differentiation of a human oligodendrocyte cell line. J Neurosci Res.

[B22] Feher LZ, Kalman J, Puskas LG, Gyulveszi G, Kitajka K, Penke B, Palotas M, Samarova EI, Molnar J, Zvara A, Matin K, Bodi N, Hugyecz M, Pakaski M, Bjelik A, Juhasz A, Bogats G, Janka Z, Palotas A (2005). Impact of haloperidol and risperidone on gene expression profile in the rat cortex. Neurochem Int.

[B23] Niederkorn JY (2006). See no evil, hear no evil, do no evil: the lessons of immune privilege. Nat Immunol.

[B24] Rothermundt M, Arolt V, Bayer TA (2001). Review of immunological and immunopathological findings in schizophrenia. Brain Behav Immun.

[B25] Sham PC, O'Callaghan E, Takei N, Murray GK, Hare EH, Murray RM (1992). Schizophrenia following pre-natal exposure to influenza epidemics between 1939 and 1960. Br J Psychiatry.

[B26] Patterson PH (2002). Maternal infection: window on neuroimmune interactions in fetal brain development and mental illness. Curr Opin Neurobiol.

[B27] Tohmi M, Tsuda N, Watanabe Y, Kakita A, Nawa H (2004). Perinatal inflammatory cytokine challenge results in distinct neurobehavioral alterations in rats: implication in psychiatric disorders of developmental origin. Neurosci Res.

[B28] Meyer U, Schwendener S, Feldon J, Yee BK (2006). Prenatal and postnatal maternal contributions in the infection model of schizophrenia. Exp Brain Res.

[B29] Battle YL, Martin BC, Dorfman JH, Miller LS (1999). Seasonality and infectious disease in schizophrenia: the birth hypothesis revisited. J Psychiatr Res.

[B30] Vestal DJ (2005). The guanylate-binding proteins (GBPs): proinflammatory cytokine-induced members of the dynamin superfamily with unique GTPase activity. J Interferon Cytokine Res.

[B31] Naschberger E, Bauer M, Sturzl M (2005). Human guanylate binding protein-1 (hGBP-1) characterizes and establishes a non-angiogenic endothelial cell activation phenotype in inflammatory diseases. Adv Enzyme Regul.

[B32] Horvath AJ, Irving JA, Rossjohn J, Law RH, Bottomley SP, Quinsey NS, Pike RN, Coughlin PB, Whisstock JC (2005). The murine orthologue of human antichymotrypsin: a structural paradigm for clade A3 serpins. J Biol Chem.

[B33] Berninger RW (1985). Protease inhibitors of human plasma. Alpha 1-antitrypsin. J Med.

[B34] Tanaka SS, Yamaguchi YL, Tsoi B, Lickert H, Tam PP (2005). IFITM/Mil/fragilis family proteins IFITM1 and IFITM3 play distinct roles in mouse primordial germ cell homing and repulsion. Dev Cell.

[B35] Lubeseder-Martellato C, Guenzi E, Jorg A, Topolt K, Naschberger E, Kremmer E, Zietz C, Tschachler E, Hutzler P, Schwemmle M, Matzen K, Grimm T, Ensoli B, Sturzl M (2002). Guanylate-binding protein-1 expression is selectively induced by inflammatory cytokines and is an activation marker of endothelial cells during inflammatory diseases. Am J Pathol.

[B36] Setzu A, Lathia JD, Zhao C, Wells K, Rao MS, Ffrench-Constant C, Franklin RJ (2006). Inflammation stimulates myelination by transplanted oligodendrocyte precursor cells. Glia.

[B37] Rhodes KE, Raivich G, Fawcett JW (2006). The injury response of oligodendrocyte precursor cells is induced by platelets, macrophages and inflammation-associated cytokines. Neuroscience.

[B38] Cabrera Gomez JA, Cordero Gutierrez JR, Fernandez Lopez O, Reyes Gutierrez B, Romero Garcia K, Simon Consuegra J, Feas Cruz R, Gonzalez Quevedo A, Alfaro Capdegille I, Del Pino Falcon M (1993). Treatment of schizophrenic disorder, paranoid type, with intramuscular recombinant alpha-2b interferon. Biotherapy.

[B39] Browning CH (1996). Nonsteroidal anti-inflammatory drugs and severe psychiatric side effects. Int J Psychiatry Med.

[B40] Skurkovich SV, Aleksandrovsky YA, Chekhonin VP, Ryabukhin IA, Chakhava KO, Skurkovich B (2003). Improvement in negative symptoms of schizophrenia with antibodies to tumor necrosis factor-alpha and to interferon-gamma: a case report. J Clin Psychiatry.

