# Supplementary material for: Inflammation-related genes up-regulated in schizophrenia brains
Source: BMC Psychiatry. 2007 Sep 6;7:46. doi: 10.1186/1471-244X-7-46 (PMC2080573; doi:10.1186/1471-244X-7-46)
Supplement: Additional file 1 — Supplementary Table 1 – Detailed description of studied subjects. Information about brain bank of origin, gender, age, time post-mortem (PMI), agonal state, age of onset, brain pH and medication for the 110 individuals included in the study. [file 1471-244X-7-46-S1.pdf]

Source	Diagnosis	GENDER	AGE	PMI	Agonal state	AGE ONSET	BRAIN pH
Stanley Brain	control	M	52	28	Cardiac		6,5
Stanley Brain	control	F	44	25	Cardiac		6,3
Stanley Brain	control	M	59	26	Cardiac		6,4
Stanley Brain	control	M	52	8	Cardiac		6,5
Stanley Brain	control	M	52	22	Cardiac		6,2
Stanley Brain	control	M	53	28	Cardiac		6,2
Stanley Brain	control	M	44	10	Cardiac		6,4
Stanley Brain	control	F	35	23	Cardiac		6,6
Stanley Brain	control	M	41	11	Pulmonary embolus		6
Stanley Brain	control	M	42	27	Cardiac		6,6
Stanley Brain	control	F	35	40	Pulmonary embolus		5,8
Stanley Brain	control	F	68	13	Pulmonary embolus		6,3
Stanley Brain	control	M	58	27	Cardiac		6
Stanley Brain	control	F	29	42	Motor vehicle accident		6,2
Stanley Brain	control	F	57	26	Motor vehicle accident		6
Stanley Brain	schizophrenia	F	30	60	Suicide: jumped	22	6,2
Stanley Brain	schizophrenia	M	52	61	Cardiac	20	6
Stanley Brain	schizophrenia	M	30	32	Pneumonia	13	5,8
Stanley Brain	schizophrenia	F	62	26	Motor vechicle accident	38	6,1
Stanley Brain	schizophrenia	F	60	40	Cardiac	15	6,2
Stanley Brain	schizophrenia	M	60	31	Accidental drowning	27	6,2
Stanley Brain	schizophrenia	M	32	19	Acute alcohol intoxicati	27	6,1
Stanley Brain	schizophrenia	M	31	14	Suicide: jumped	18	5,8
Stanley Brain	schizophrenia	F	58	26	Cardiac	42	5,9
Stanley Brain	schizophrenia	M	25	32	Suicide: hanged	20	6,6

Source	Diagnosis	GENDER	AGE	PMI	Agonal state	AGE ONSE	BRAIN pH
Stanley Brain	schizophrenia	M	44	50	Cardiac	17	6,5
Stanley Brain	schizophrenia	M	44	29	Pulmonary disease	21	5,9
Stanley Brain	schizophrenia	F	56	12	Suicide: overdose	24	6,4
Stanley Brain	schizophrenia	M	35	35	Cardiac	19	6,5
Stanley Brain	schizophrenia	F	49	38	Cardiac	25	6,2
Harvard Brain	control	M	66	23			
Harvard Brain	control	M	24	13			
Harvard Brain	control	M	81	19			
Harvard Brain	control	F	46	23			
Harvard Brain	control	F	50	21			
Harvard Brain	control	M	66	19			
Harvard Brain	control	M	40	28			
Harvard Brain	control	M	48	15			
Harvard Brain	control	M	44	23			
Harvard Brain	control	M	47	19			
Harvard Brain	control	M	38	22			
Harvard Brain	control	M	85	14			
Harvard Brain	control	M	69	9,2			
Harvard Brain	control	F	69	21			
Harvard Brain	control	F	44	11			
Harvard Brain	control	F	75	26			
Harvard Brain	schizophrenia	F	84	12			
Harvard Brain	schizophrenia	F	82	20			
Harvard Brain	schizophrenia	F	66	7,2			
Harvard Brain	schizophrenia	M	44	18			
Harvard Brain	schizophrenia	F	49	29			
Harvard Brain	schizophrenia	F	74	28			

Source	Diagnosis	GENDER	AGE	PMI	Agonal state	AGE ONSE	BRAIN pH
Harvard Brain	schizophrenia	M	66	21			
Harvard Brain	schizophrenia	M	69	18			
Harvard Brain	schizophrenia	M	42	14			
Harvard Brain	schizophrenia	M	46	30			
Harvard Brain	schizophrenia	M	44	19			
Harvard Brain	schizophrenia	M	35	28			
Harvard Brain	schizophrenia	M	46	19			
Harvard Brain	schizophrenia	M	26	16			
Harvard Brain	schizophrenia	M	47	19			
Harvard Brain	schizophrenia	F	67	22			
Maudsley Inst	control	M	57	21	Mycocardial Infarction		
Maudsley Inst	control	M	78	25	Coronary Artery Occlusion		
Maudsley Inst	control	M	63	26	Coronary Artery Occlusion		
Maudsley Inst	control	F	71	30	Pulmonary Embolism		
Maudsley Inst	control	M	75	85	N/A		
Maudsley Inst	control	F	86	70	Peritonitis		
Maudsley Inst	control	M	56	51	Gastrointestinal Bleed		
Maudsley Inst	control	M	77	96	Mycocardial Infarction		
Maudsley Inst	control	F	62	81	Haemathorax		
Maudsley Inst	control	M	69	52	Aortic Aneurism		
Maudsley Inst	control	M	49	44	Pulmonary Oedema		
Maudsley Inst	control	F	73	70	Pelvic carcinoma		
Maudsley Inst	control	F	96	16	Chronic Ischaemic heart disease		
Maudsley Inst	control	F	82	48	Haemopericardium		
Maudsley Inst	control	F	80	31	Left Ventricular Failure		
Maudsley Inst	control	M	64	48	Pulmonary Oedema		
Maudsley Inst	control	F	80	31	Pulmonary Embolism		

Source	Diagnosis	GENDER	AGE	PMI	Agonal state	AGE ONSE	BRAIN pH
Maudsley Inst	control	M	21	37	Sudden accident		
Maudsley Inst	control	F	20	38	Sudden accident		
Maudsley Inst	control	F	75	51	Pulmonary Infarction		
Maudsley Inst	control	F	71	91	Cardiorespiratory failure		
Maudsley Inst	control	M	48	60	Ruptured Aortic Aneurism		
Maudsley Inst	control	M	62	91	Ischaemic heart disease		
Maudsley Inst	control	F	90	30	Mycocardial Infarction		
Maudsley Inst	schizophrenia	M	70	48	Bronchopneumonia		
Maudsley Inst	schizophrenia	F	75	44	Bronchopneumonia		
Maudsley Inst	schizophrenia	M	64	84	Pulmonary Embolus		
Maudsley Inst	schizophrenia	F	71	67	Pulmonary Embolus		
Maudsley Inst	schizophrenia	F	70	100	Bronchopneumonia		
Maudsley Inst	schizophrenia	F	87	7	Bronchopneumonia		
Maudsley Inst	schizophrenia	M	46	38	Multiple injuries		
Maudsley Inst	schizophrenia	F	69	24	N/A		
Maudsley Inst	schizophrenia	M	62	36	Pulmonary tuberculosis		
Maudsley Inst	schizophrenia	F	76	97	Bronchopneumonia		
Maudsley Inst	schizophrenia	M	67	24	Mycocardial Infarction		
Maudsley Inst	schizophrenia	M	49	24	Mycocardial Infarction		
Maudsley Inst	schizophrenia	F	49	24	Perforatyed duodenal		
Maudsley Inst	schizophrenia	M	31	27	Multiple organ failure		
Maudsley Inst	schizophrenia	F	34	23	Acute Pulmonary oedema		
Maudsley Inst	schizophrenia	M	51	44	Mycocardial Infarction		
Maudsley Inst	schizophrenia	M	64	48	Acute Liver failure		
Maudsley Inst	schizophrenia	F	84	42	Bronchopneumonia		
Maudsley Inst	schizophrenia	M	45	45	Intra-abominal sepsis		
Maudsley Inst	schizophrenia	M	87	48	Bronchopneumonia		

Source	Diagnosis	GENDER	AGE	PMI	Agonal state	AGE ONSE	BRAIN pH
Maudsley Inst	schizophrenia	F	32	22	Pulmonary Embolus		
Maudsley Inst	schizophrenia	M	35	67	Bronchopneumonia		
Maudsley Inst	schizophrenia	F	75	50	Cardiovascular accident		
Maudsley Inst	schizophrenia	M	62	48	Ischaemic heart disease		

CNS MEDS AT DEATH	CN	CNS	SUBSTANCE ABUSE
None			Alcohol abuse when younger but abstinent last 8 years. No use of drugs.
None			Alcohol use light; Occasional marihuana in younger years.
None			2-3 beers each night but caused no problems and liver unremarkable; No use
None			Alcohol use light; No use of drugs.
None			4-6 beers /day but never missed work and liver unremarkable. No use of dru
None			Alcohol abuse in 20s but abstinent for over 30 years. In 20s tried marihuana
None			Alcohol use light; No use of drugs.
None			Alcohol use light; No use of drugs.
None			Alcohol use light; Used marihuana "a few times" in college.
None			Alcohol use light; No use of drugs.
None			Alcohol use light; No use of drugs.
None			Alcohol use light; No use of drugs.
None			2 beers /day; No use of drugs.
Saw counselor for weight control			Alcohol use light; no use of drugs.
None			Alcohol use light; No use of drugs.
Thiothixene, desipramine			Marihuana abuse.
None; untreated for over 20 yrs.			No use of alcohol; No use of drugs.
Risperidone,thioridazine			Moderate use of alcohol; No use of drugs.
None; untreated for several months			Light use of alcohol; No use of drugs.
None; had ECT but probably never treated other			No use of alcohol; No use of drugs.
Thioridazine, amitriptyline			No use of alcohol; No use of drugs.
Clozapine			Alcohol abuse; Amphetamine abuse.
Clozapine			Light use of alcohol; No use of drugs.
Haloperidol, iphenhydramine			Past alcohol abuse; No use of drugs.
Risperidone, paroxetine			Past alcohol abuse and marihuana use.

CNS MEDS AT DEATH	CN	CNS	SUBSTANCE ABUSE
Haloperidol, carbamazepine, fluoxetine, clonazep			Moderate alcohol use; Little or no use of drugs.
Clozapine, chlorpromazine, lithium			Alcohol dependence; Past polysubstance abuse.
Haloperidol, lithium, diphenhydramine, chloral hy			No alcohol use; Occasional past marihuana use.
Clozapine, chlorpromazine, maprotiline, benzotro			Moderate alcohol use; Polysubstance abuse.
Halperidol, clozapine, clonazepam			No use of alcohol or drugs.

CNS MEDS AT DEATH	CN	CNS	SUBSTANCE ABUSE
none			
none			
none			
none			
none			
none			
none			
none			
none			
none			
none			
none			
none			
none			
none			
none			
none			

CNS MEDS AT DEATH	CN	CN	SUBSTANCE ABUSE
none			
none			
none			
none			
none			
none			
none			
PROMAZINE			
CHLORPROMAZINE	O		
none			
CHLORPROMAZINE	E		
TRIFLUOPERAZINE	O	TR	
none			
SULPIRIDE			
none			
none			
HALOPERIDOL			
STELAZINE	SI	GA	
CHLORPROMAZINE	U	DE	
none			
CHLORPROMAZINE			
NA, data not available			
THIORIDAZINE	U		
none			
none			
NA, data not available			
STELAZINE	O		

CNS MEDS AT DEATH	CN	CNS	SUBSTANCE ABUSE
CHLORPROMAZINE	U		
TRIFLUOPERAZINE	U		
TRIFLUOPERAZINE			
none			

smoker
NS
S
S
NS
S
NS
U
U
U
U
U
NS
U
U
U
S
U
NS
NS
U
U
S
NS
NS
S

smoker
S
S
S
NS
S

smoker

smoker

smoker
