# Supplementary material for: Inflammation-related genes up-regulated in schizophrenia brains
Source: BMC Psychiatry. 2007 Sep 6;7:46. doi: 10.1186/1471-244X-7-46 (PMC2080573; doi:10.1186/1471-244X-7-46)
Supplement: Additional file 2 — Supplementary Table 2 – Genes differentially expressed in schizophrenic subjects not treated at death. List of 29 clones showing evidence of being differentially expressed in frontal cortex autopsy samples from schizophrenic subjects as compared to unaffected individuals. Results from experiments of hybridizing pooled mRNA samples to cDNA microarrays. [file 1471-244X-7-46-S2.doc]

**Supplementary tables 2,3 and 4:**

| Table 1. Experimental design and model parameterization |
| --- |
| |  |  |  |  | |  |  | | | | | --- | --- | --- | --- | --- | --- | --- | --- | --- | --- | |  |  |  |  |  |  |  |  |  |  | |  |  |  |  |  |  |  |  |  |  | |  |  |  |  |  |  |  |  |  |  | |  |  |  |  |  |  |  |  |  |  | |  |  |  |  |  |  |  |  |  |  | |  |  |  |  |  |  |  |  |  |  | |  |  |  |  |  |  |  |  |  |  | |  |  |  |  |  |  |  |  |  |  | |  |  |  |  |  |  |  |  |  |  | |  |  |  |  |  |  |  |  |  |  | |  |  |  |  |  |  |  |  |  |  | |  |  |  |  |  |  |  |  |  |  | |  |  |  |  |  |  |  |  |  |  | |  |  |  |  |  |  |  |  |  |  | |  |  |  |  |  |  |  |  |  |  | |  |  |  |  |  |  |  |  |  |  | |  |  |  |  |  |  |  |  |  |  | |

|  |
| --- |
|  |

|  |  |
| --- | --- |
|  | |

|  |
| --- |
|  |

|  |
| --- |
|  |

|  |  |
| --- | --- |
|  |  |

| Table 2. Earlier reported genes present in the differentially expressed gene list. |
| --- |
| |  |  |  |  |  |  |  |  |  |  | | --- | --- | --- | --- | --- | --- | --- | --- | --- | --- | |  |  |  |  |  |  |  |  |  | |  |  |  |  |  |  |  |  |  | |  |  |  |  |  |  |  |  |  | |  |  |  |  |  |  |  |  |  | |  |  |  |  |  |  |  |  |  | |  |  |  |  |  |  |  |  |  | |  |  |  |  |  |  |  |  |  | |  |  |  |  |  |  |  |  |  | |  |  |  |  |  |  |  |  |  | |  |  |  |  |  |  |  |  |  | |  |  |  |  |  |  |  |  |  | |  |  |  |  |  |  |  |  |  | |

| . |
| --- |
| |  |  |  |  |  |  |  |  |  |  | | --- | --- | --- | --- | --- | --- | --- | --- | --- | --- | |  |  |  |  |  |  |  |  |  | |  |  |  |  |  |  |  |  |  | |  |  |  |  |  |  |  |  |  | |  |  |  |  |  |  |  |  |  | |

| Table 4 Genes from selected pathways present in the differentially expressed gene list. |
| --- |
| |  |  |  |  |  |  |  | | --- | --- | --- | --- | --- | --- | --- | |  |  |  |  |  |  | |  |  |  |  |  |  | |  |  |  |  |  |  | |  |  |  |  |  |  | |  |  |  |  |  |  | |  |  |  |  |  |  | |  |  |  |  |  |  | |  |  |  |  |  |  |  | |  |  |  |  |  |  | |  |  |  |  |  |  | |  |  |  |  |  |  | |  |  |  |  |  |  | |  |  |  |  |  |  | |  |  |  |  |  |  |  | |  |  |  |  |  |  | |  |  |  |  |  |  | |  |  |  |  |  |  | |  |  |  |  |  |  | |  |  |  |  |  |  | |  |  |  |  |  |  | |  |  |  |  |  |  | |  |  |  |  |  |  | |  |  |  |  |  |  |  | |  |  |  |  |  |  | |  |  |  |  |  |  | |  |  |  |  |  |  | |

|  |
| --- |
|  |

|  |
| --- |
|  |

|  |
| --- |
|  |

| Table 6. RT-PCR estimates and F values of the 8 genes. Significant F values are indicated by; * p<0.05 or **p<0.01. |
| --- |
| |  |  | | |  | | | --- | --- | --- | --- | --- | --- | |  |  |  |  |  |  | |  |  |  |  |  |  | |  |  |  |  |  |  | |  |  |  |  |  |  | |  |  |  |  |  |  | |  |  |  |  |  |  | |  |  |  |  |  |  | |  |  |  |  |  |  | |  |  |  |  |  |  | |

|  |
| --- |
|  |

|  |  |
| --- | --- |
|  |  |
|  | |

**Supplementary table 2.**

Genes differentially expressed in untreated patients compared to controls. List ordered by decreased PenF value.

| Symbol | CLONEID | Atyp  Estimate | Typ  Estimate | NoDrug  Estimate | Uknw  Estimate | Atyp  Std Error | Typ  Std Error | NoDrug  Std Error | Uknw  Std Error | PenF |
| --- | --- | --- | --- | --- | --- | --- | --- | --- | --- | --- |
| SERPINA3 | 450533 | 2,968636 | 0,202457 | 2,169048 | 3,547087 | 0,142677 | 0,142677 | 0,142677 | 0,472993 | 72,63269 |
| IFITM3 | 809910 | 0,852469 | 0,656203 | 2,120235 | 1,864168 | 0,186484 | 0,186484 | 0,186484 | 0,618217 | 56,76417 |
| FOS | 811015 | 0,188043 | 0,047509 | 0,963668 | 2,674425 | 0,10059 | 0,10059 | 0,10059 | 0,33347 | 17,02819 |
| C10orf10 | 135811 | 1,21546 | -0,5856 | 1,10344 | 1,289525 | 0,198318 | 0,198318 | 0,198318 | 0,657448 | 14,53864 |
| IFITM2 | 1592837 | 1,055751 | 0,630075 | 1,426945 | 2,08602 | 0,310583 | 0,310583 | 0,310583 | 1,029621 | 14,45325 |
| CLIPR-59 | 173145 | -0,27238 | -0,10364 | -1,06574 | -0,66648 | 0,185931 | 0,185931 | 0,185931 | 0,616386 | 14,37944 |
| TF | 212429 | -1,40628 | 0,833242 | -1,03522 | -0,79566 | 0,18617 | 0,18617 | 0,18617 | 0,617175 | 13,55237 |
| IFITM2 | 1455976 | 1,203623 | 0,533496 | 1,17832 | 2,828528 | 0,241658 | 0,241658 | 0,241658 | 0,801125 | 13,50405 |
| No symbol | 1569263 | -0,29067 | 0,433157 | 1,139081 | -0,05615 | 0,231604 | 0,231604 | 0,231604 | 0,767795 | 13,23197 |
| No symbol | 324180 | -0,36206 | 0,441261 | 1,092196 | 1,04526 | 0,246487 | 0,246487 | 0,246487 | 0,817135 | 11,34209 |
| APP | 323371 | -0,73303 | -0,84357 | -1,54616 | -0,0406 | 0,409155 | 0,409155 | 0,409155 | 1,356398 | 11,2858 |
| VAMP2 | 179022 | 0,147294 | -0,0505 | -0,78027 | -0,41934 | 0,103239 | 0,103239 | 0,103239 | 0,34225 | 11,05416 |
| EHD3 | 1468655 | -0,00859 | -0,3514 | -1,02037 | -0,46836 | 0,237271 | 0,237271 | 0,237271 | 0,786581 | 10,33763 |
| DNCI1 | 39189 | 0,384119 | 0,081629 | -0,79515 | -0,83299 | 0,132941 | 0,132941 | 0,132941 | 0,440715 | 10,18287 |
| No symbol | 176572 | -0,28346 | -0,3994 | -0,89039 | 0,188696 | 0,183219 | 0,183219 | 0,183219 | 0,607392 | 10,16575 |
| ELL | 44310 | -1,64878 | 0,404995 | -0,90137 | -1,24094 | 0,189228 | 0,189228 | 0,189228 | 0,627314 | 10,12739 |
| GBP1 | 841008 | -0,06162 | 0,795036 | 0,992271 | 1,252936 | 0,232339 | 0,232339 | 0,232339 | 0,770232 | 10,00618 |
| PCTK3 | 725677 | -1,06327 | 0,317696 | -0,84635 | -0,35925 | 0,169103 | 0,169103 | 0,169103 | 0,560596 | 9,810697 |
| GRSF1 | 840384 | 0,194444 | 0,073137 | 0,859256 | 0,897568 | 0,182641 | 0,182641 | 0,182641 | 0,605476 | 9,492953 |
| No symbol | 274677 | -0,21995 | 0,225454 | 0,853259 | 1,466447 | 0,198329 | 0,198329 | 0,198329 | 0,657487 | 8,692875 |
| RGS4 | 429349 | 0,504992 | -0,02078 | -0,72715 | -0,55567 | 0,152587 | 0,152587 | 0,152587 | 0,505846 | 7,810068 |
| GFAP | 382693 | -0,15908 | 0,399677 | 0,843127 | -0,21801 | 0,216818 | 0,216818 | 0,216818 | 0,718778 | 7,775111 |
| No symbol | 207558 | 0,895188 | 0,442838 | -0,78303 | -2,30632 | 0,186078 | 0,186078 | 0,186078 | 0,616871 | 7,756947 |
| RTN3 | 380851 | -0,20394 | 0,012142 | -0,69803 | -0,56471 | 0,138407 | 0,138407 | 0,138407 | 0,458837 | 7,664226 |
| SCN3B | 39815 | 0,334134 | -0,20467 | -0,71092 | -1,64508 | 0,148656 | 0,148656 | 0,148656 | 0,492811 | 7,598325 |
| No symbol | 310356 | -0,07847 | 0,014693 | 0,744276 | 0,994395 | 0,169391 | 0,169391 | 0,169391 | 0,561553 | 7,57674 |
| UNC5A | 166871 | 0,0999 | -0,43334 | -0,71021 | 1,002494 | 0,154075 | 0,154075 | 0,154075 | 0,510779 | 7,400612 |
| CDK5R1 | 796579 | 0,575047 | -0,47217 | -0,62587 | 0,525422 | 0,09369 | 0,09369 | 0,09369 | 0,310592 | 7,363551 |
| TDE1 | 859912 | 0,15019 | 0,147273 | -0,74313 | -0,82947 | 0,182689 | 0,182689 | 0,182689 | 0,605637 | 7,098854 |

**Supplementary table 3****.**

Genes differentially expressed in patients treated with atypical neuroleptics compared to controls. List ordered by decreased PenF value.

| Symbol | CLONEID | Atyp  Estimate | Typ  Estimate | NoDrug  Estimate | Uknw  Estimate | Atyp  Std Error | Typ  Std Error | NoDrug  Std Error | Uknw  Std Error | PenF |
| --- | --- | --- | --- | --- | --- | --- | --- | --- | --- | --- |
| SERPINA3 | 450533 | 2,968636 | 0,202457 | 2,169048 | 3,547087 | 0,142677 | 0,142677 | 0,142677 | 0,472993 | 136,0529 |
| SCD | 810711 | -1,72341 | 0,313398 | -0,58389 | -0,12368 | 0,159382 | 0,159382 | 0,159382 | 0,528371 | 42,53984 |
| MAG | 32444 | -1,7636 | 0,425035 | -0,55883 | -1,69059 | 0,21091 | 0,21091 | 0,21091 | 0,699192 | 34,98613 |
| No symbol | 840708 | 1,593958 | -0,06102 | 0,214469 | 0,022331 | 0,171897 | 0,171897 | 0,171897 | 0,569859 | 34,34937 |
| ELL | 44310 | -1,64878 | 0,404995 | -0,90137 | -1,24094 | 0,189228 | 0,189228 | 0,189228 | 0,627314 | 33,88559 |
| CRYAB | 839736 | -1,33677 | 0,536931 | -0,326 | 0,831571 | 0,137473 | 0,137473 | 0,137473 | 0,455741 | 28,22235 |
| KIAA1394 | 1593420 | -1,37474 | 0,567781 | -0,66003 | -1,33421 | 0,153278 | 0,153278 | 0,153278 | 0,508137 | 27,82856 |
| FA2H | 811891 | -1,17265 | 0,439067 | -0,33568 | -0,16061 | 0,086444 | 0,086444 | 0,086444 | 0,286572 | 26,50016 |
| PLEKHB1 | 447416 | -1,21649 | 0,569801 | 0,148868 | 0,671133 | 0,120149 | 0,120149 | 0,120149 | 0,398309 | 25,14433 |
| No symbol | 124661 | -2,63384 | -0,99576 | 0,448045 | 4,031274 | 0,481763 | 0,481763 | 0,481763 | 1,597103 | 25,08772 |
| K5B | 377827 | -1,50504 | -1,13555 | 0,137698 | 3,161412 | 0,21425 | 0,21425 | 0,21425 | 0,710264 | 25,07881 |
| TF | 212429 | -1,40628 | 0,833242 | -1,03522 | -0,79566 | 0,18617 | 0,18617 | 0,18617 | 0,617175 | 25,00882 |
| PLXNB3 | 282500 | -1,26668 | 0,452084 | -0,28473 | -1,30399 | 0,148141 | 0,148141 | 0,148141 | 0,491106 | 24,17692 |
| GSN | 214990 | -1,1269 | 0,278668 | -0,39672 | -0,29454 | 0,093756 | 0,093756 | 0,093756 | 0,310814 | 23,86655 |
| PEX7 | 2018758 | -1,21403 | 0,271507 | -0,21933 | 0,223116 | 0,132101 | 0,132101 | 0,132101 | 0,437931 | 23,82268 |
| APOE | 83995 | -1,23823 | -0,03001 | -0,30064 | -0,05246 | 0,150609 | 0,150609 | 0,150609 | 0,499288 | 22,84913 |
| CDC14B | 781061 | -1,27439 | 0,669069 | 0,173997 | 1,372329 | 0,174788 | 0,174788 | 0,174788 | 0,579443 | 21,66325 |
| C10orf10 | 245774 | 1,144133 | -0,16461 | 0,455519 | 1,332421 | 0,128618 | 0,128618 | 0,128618 | 0,426383 | 21,47359 |
| GJB1 | 288663 | -1,13924 | 0,289033 | -0,43988 | -0,15579 | 0,132318 | 0,132318 | 0,132318 | 0,43865 | 20,95845 |
| BM039 | 970649 | -1,5318 | -0,70264 | -0,04645 | 2,589579 | 0,27248 | 0,27248 | 0,27248 | 0,903304 | 19,77378 |
| AGXT2L1 | 363007 | -1,31201 | -0,34315 | -0,31964 | -0,54759 | 0,206657 | 0,206657 | 0,206657 | 0,685093 | 19,75746 |
| HIPK2 | 950603 | -1,62856 | 0,958448 | 0,439863 | 1,028729 | 0,301774 | 0,301774 | 0,301774 | 1,000418 | 19,57568 |
| OLIG1 | 41214 | -1,07698 | 0,3683 | -0,20033 | 0,5299 | 0,123883 | 0,123883 | 0,123883 | 0,410688 | 19,4075 |
| PLXNB3 | 36573 | -1,35847 | 0,050755 | -0,33995 | 0,094235 | 0,225515 | 0,225515 | 0,225515 | 0,747611 | 19,3696 |
| PLEKHB1 | 1471451 | -1,19181 | 0,353326 | -0,07568 | 0,84826 | 0,170056 | 0,170056 | 0,170056 | 0,563758 | 19,36822 |
| KIAA1189 | 36462 | -1,23327 | 0,514716 | -0,299 | -0,48103 | 0,188676 | 0,188676 | 0,188676 | 0,625484 | 19,0079 |
| THBD | 205185 | -0,95849 | 0,474054 | -0,201 | -1,80367 | 0,075218 | 0,075218 | 0,075218 | 0,249358 | 18,34608 |
| C10orf10 | 137554 | 1,13746 | -0,3843 | 0,264903 | 0,817524 | 0,164626 | 0,164626 | 0,164626 | 0,545755 | 18,09034 |
| No symbol | 180902 | -1,1908 | 0,662099 | -0,42141 | -0,53246 | 0,185927 | 0,185927 | 0,185927 | 0,616372 | 17,95237 |
| C10orf10 | 135811 | 1,21546 | -0,5856 | 1,10344 | 1,289525 | 0,198318 | 0,198318 | 0,198318 | 0,657448 | 17,64035 |
| No symbol | 897252 | -0,96624 | 0,412646 | 0,118713 | -1,46685 | 0,093799 | 0,093799 | 0,093799 | 0,310956 | 17,54376 |
| MOBP | 51974 | -1,17912 | 0,606532 | -0,15935 | -0,4682 | 0,197911 | 0,197911 | 0,197911 | 0,6561 | 16,63339 |
| No symbol | 66714 | -1,11058 | 0,717805 | 0,321099 | -0,76608 | 0,175596 | 0,175596 | 0,175596 | 0,582123 | 16,39005 |
| No symbol | 1609795 | 1,018048 | -0,23206 | -0,09035 | 0,706496 | 0,139804 | 0,139804 | 0,139804 | 0,463468 | 16,20341 |
| TNFAIP2 | 810444 | 0,959473 | 0,00928 | -0,04144 | 0,102097 | 0,117055 | 0,117055 | 0,117055 | 0,388053 | 15,83947 |
| No symbol | 184367 | -1,19345 | 0,040143 | -0,59126 | -0,42625 | 0,216954 | 0,216954 | 0,216954 | 0,71923 | 15,56849 |
| PLXNB1 | 755952 | -0,97673 | 0,184329 | -0,01866 | 0,454201 | 0,130821 | 0,130821 | 0,130821 | 0,433687 | 15,50419 |
| PCTK3 | 725677 | -1,06327 | 0,317696 | -0,84635 | -0,35925 | 0,169103 | 0,169103 | 0,169103 | 0,560596 | 15,48405 |
| RTKN | 345128 | -0,99375 | 0,374049 | -0,41809 | -0,4909 | 0,139937 | 0,139937 | 0,139937 | 0,463909 | 15,43026 |
| TNKS | 453193 | -1,04076 | 0,682355 | -0,1836 | -0,77099 | 0,16341 | 0,16341 | 0,16341 | 0,541725 | 15,23003 |
| No symbol | 809838 | -0,91786 | 0,393897 | -0,11278 | -0,16562 | 0,106899 | 0,106899 | 0,106899 | 0,354384 | 15,08561 |
| C9orf28 | 51860 | -0,90169 | 0,36583 | -0,27202 | 0,041302 | 0,0994 | 0,0994 | 0,0994 | 0,329522 | 14,97375 |
| CDC42EP1 | 159462 | -1,04229 | 0,399883 | -0,41055 | -1,09013 | 0,177817 | 0,177817 | 0,177817 | 0,589484 | 14,28731 |
| IFITM2 | 1455976 | 1,203623 | 0,533496 | 1,17832 | 2,828528 | 0,241658 | 0,241658 | 0,241658 | 0,801125 | 14,09025 |
| TTYH2 | 770878 | -1,00177 | 0,269184 | -0,19763 | -0,49252 | 0,171769 | 0,171769 | 0,171769 | 0,569437 | 13,57557 |
| PLEKHB1 | 731469 | -1,12229 | 0,531146 | 0,398491 | 0,780106 | 0,220695 | 0,220695 | 0,220695 | 0,731631 | 13,5253 |
| SCD | 1616241 | -0,94002 | -0,09332 | -0,27314 | 0,460613 | 0,145373 | 0,145373 | 0,145373 | 0,48193 | 13,4801 |
| No symbol | 418129 | -0,84964 | -0,23229 | -0,02134 | 0,178467 | 0,097485 | 0,097485 | 0,097485 | 0,323175 | 13,38792 |
| No symbol | 31056 | -0,93391 | -0,11295 | -0,19528 | 0,117497 | 0,144622 | 0,144622 | 0,144622 | 0,479441 | 13,34973 |
| OLIG2 | 26884 | -0,90019 | 0,666207 | -0,25834 | -0,39787 | 0,131829 | 0,131829 | 0,131829 | 0,43703 | 13,11308 |
| LOC23117 | 785605 | -0,88588 | -0,30034 | -0,20351 | 0,826329 | 0,125268 | 0,125268 | 0,125268 | 0,415279 | 13,0557 |
| RNASE1 | 840493 | -0,90456 | 0,672175 | -0,33699 | -1,57397 | 0,136144 | 0,136144 | 0,136144 | 0,451335 | 12,99743 |
| GPRC5B | 321580 | -1,24693 | 0,345542 | -0,41878 | -0,00719 | 0,279959 | 0,279959 | 0,279959 | 0,928099 | 12,66205 |
| MGC35048 | 33603 | -1,05182 | 0,114312 | -0,46318 | 0,809756 | 0,20902 | 0,20902 | 0,20902 | 0,692928 | 12,55664 |
| TP53INP2 | 366167 | -0,87963 | 0,507009 | -0,16053 | 0,610467 | 0,134369 | 0,134369 | 0,134369 | 0,445449 | 12,3853 |
| KCNJ10 | 30028 | -1,11475 | 0,397625 | -0,34517 | 0,441688 | 0,237043 | 0,237043 | 0,237043 | 0,785827 | 12,35157 |
| No symbol | 462412 | -1,03583 | 0,291132 | -0,30112 | 0,001229 | 0,207474 | 0,207474 | 0,207474 | 0,687803 | 12,26723 |
| COL4A5 | 42864 | -0,78333 | 0,192965 | -0,03167 | -0,54254 | 0,075651 | 0,075651 | 0,075651 | 0,250791 | 12,23763 |
| HIPK2 | 1751004 | -0,97547 | 0,195566 | -0,12116 | 0,451389 | 0,186329 | 0,186329 | 0,186329 | 0,617704 | 12,02406 |
| GBP2 | 298899 | 0,888744 | 0,333294 | 0,087855 | -0,30897 | 0,151218 | 0,151218 | 0,151218 | 0,501307 | 11,73912 |
| No symbol | 1948645 | -1,63414 | -0,85478 | -0,07259 | 2,070972 | 0,441702 | 0,441702 | 0,441702 | 1,464295 | 11,14914 |
| No symbol | 284670 | -0,89547 | -0,03482 | -0,21925 | 0,794976 | 0,166625 | 0,166625 | 0,166625 | 0,552382 | 11,10889 |
| CNTN2 | 28510 | -1,0311 | 0,446404 | -0,49862 | -0,49859 | 0,228487 | 0,228487 | 0,228487 | 0,757462 | 11,00313 |
| AGT | 2019101 | -0,83785 | 0,187679 | 0,0801 | 0,475646 | 0,141417 | 0,141417 | 0,141417 | 0,468816 | 10,89776 |
| No symbol | 824179 | -1,20307 | 0,112188 | -0,17891 | -0,12446 | 0,297448 | 0,297448 | 0,297448 | 0,986076 | 10,89128 |
| VEZATIN | 773242 | -1,0474 | 0,589399 | 0,228003 | 0,524923 | 0,240394 | 0,240394 | 0,240394 | 0,796936 | 10,73346 |
| No symbol | 135897 | 0,763454 | -0,15128 | 0,426711 | 1,243347 | 0,100772 | 0,100772 | 0,100772 | 0,334073 | 10,68041 |
| ALS2CR19 | 810954 | -1,04061 | -0,51454 | 0,126138 | 1,889595 | 0,241731 | 0,241731 | 0,241731 | 0,801369 | 10,52841 |
| STX3A | 2012523 | 1,329629 | 0,058293 | 0,147445 | 0,052458 | 0,351973 | 0,351973 | 0,351973 | 1,166835 | 10,50434 |
| SOX3 | 2019116 | -0,76806 | 0,287586 | 0,013692 | 0,339415 | 0,108547 | 0,108547 | 0,108547 | 0,359848 | 10,49663 |
| RPS4Y1 | 309449 | 0,824309 | 0,191967 | -0,16191 | -0,21094 | 0,143166 | 0,143166 | 0,143166 | 0,474612 | 10,46741 |
| SLC1A2 | 52990 | -1,3967 | 0,792097 | -0,06051 | -0,01518 | 0,378633 | 0,378633 | 0,378633 | 1,255215 | 10,38858 |
| LEPREL1 | 1472797 | -0,95264 | -0,31085 | 0,132483 | 1,885059 | 0,208255 | 0,208255 | 0,208255 | 0,690392 | 10,33766 |
| No symbol | 207558 | 0,895188 | 0,442838 | -0,78303 | -2,30632 | 0,186078 | 0,186078 | 0,186078 | 0,616871 | 10,13831 |
| ITPK1 | 1563144 | -0,78188 | 0,205181 | -0,05086 | 1,028169 | 0,126294 | 0,126294 | 0,126294 | 0,418679 | 10,12684 |
| KCNJ10 | 45417 | -0,87586 | 0,204879 | -0,19679 | 0,330347 | 0,177268 | 0,177268 | 0,177268 | 0,587666 | 10,11476 |
| No symbol | 43966 | -0,9485 | 0,274297 | -0,04313 | 0,139923 | 0,211393 | 0,211393 | 0,211393 | 0,700793 | 10,09661 |
| FOXM1 | 564803 | -0,918 | -0,01034 | -0,13976 | -0,46606 | 0,197781 | 0,197781 | 0,197781 | 0,655667 | 10,08822 |
| No symbol | 49315 | -0,88805 | 0,28743 | -0,33818 | 0,788583 | 0,185044 | 0,185044 | 0,185044 | 0,613445 | 10,02585 |
| MYO1D | 1534719 | -0,85096 | 0,350074 | -0,14726 | -0,66283 | 0,169281 | 0,169281 | 0,169281 | 0,561189 | 9,909578 |
| COL16A1 | 488258 | -0,85375 | -0,20589 | -0,07458 | 0,226486 | 0,172421 | 0,172421 | 0,172421 | 0,571596 | 9,83044 |
| CDK5R2 | 1751068 | -0,94183 | 0,287952 | -0,37337 | 0,725062 | 0,214078 | 0,214078 | 0,214078 | 0,709696 | 9,828935 |
| SNRP70 | 124261 | -0,93423 | 0,682693 | -0,56348 | -1,50453 | 0,213554 | 0,213554 | 0,213554 | 0,707958 | 9,695176 |
| EPHX1 | 1601979 | -0,93553 | 0,086306 | 0,025186 | 0,266972 | 0,214223 | 0,214223 | 0,214223 | 0,710177 | 9,691184 |
| REST | 135538 | -0,82159 | 0,477307 | -0,37439 | -1,08251 | 0,160051 | 0,160051 | 0,160051 | 0,530588 | 9,638212 |
| IFITM3 | 809910 | 0,852469 | 0,656203 | 2,120235 | 1,864168 | 0,186484 | 0,186484 | 0,186484 | 0,618217 | 9,176214 |
| BCAN | 32687 | -0,87253 | 0,148251 | -0,44523 | 0,696778 | 0,196579 | 0,196579 | 0,196579 | 0,651684 | 9,1657 |
| LHPP | 279977 | -0,83975 | 0,923486 | 0,450071 | -0,12908 | 0,181472 | 0,181472 | 0,181472 | 0,601603 | 9,116696 |
| No symbol | 744436 | -0,81918 | 0,581677 | 0,209237 | 0,459748 | 0,170943 | 0,170943 | 0,170943 | 0,566696 | 9,112641 |
| SDC4 | 504763 | -0,86647 | 0,207261 | 0,111172 | 0,994073 | 0,196853 | 0,196853 | 0,196853 | 0,65259 | 9,027018 |
| CKLFSF5 | 786596 | -0,83369 | 0,096063 | -0,16192 | -0,67224 | 0,184273 | 0,184273 | 0,184273 | 0,610886 | 8,868188 |
| PPP1R1B | 277173 | -1,0047 | -0,12507 | 0,745673 | 1,425549 | 0,263725 | 0,263725 | 0,263725 | 0,874282 | 8,857 |
| No symbol | 47565 | -0,82148 | 0,566333 | -0,17244 | -1,38028 | 0,180582 | 0,180582 | 0,180582 | 0,598651 | 8,760898 |
| TTLL4 | 839770 | -1,0435 | -0,10675 | 0,090943 | 0,703264 | 0,283667 | 0,283667 | 0,283667 | 0,940393 | 8,71914 |
| SFRS11 | 204755 | -0,85172 | -0,01498 | 0,034125 | 0,005691 | 0,19699 | 0,19699 | 0,19699 | 0,653045 | 8,716669 |
| DAAM2 | 753248 | -0,72933 | 0,259248 | 0,017185 | 0,276494 | 0,12967 | 0,12967 | 0,12967 | 0,429872 | 8,686852 |
| No symbol | 28988 | 0,700061 | -0,28011 | 0,05415 | 0,397957 | 0,111635 | 0,111635 | 0,111635 | 0,370083 | 8,616099 |
| PPP1R14A | 809611 | -0,73055 | 0,278391 | 0,182521 | -0,51438 | 0,135865 | 0,135865 | 0,135865 | 0,450408 | 8,487997 |
| SFI1 | 418019 | -0,70538 | 0,240675 | -0,08233 | -0,16664 | 0,119555 | 0,119555 | 0,119555 | 0,396341 | 8,474698 |
| MALAT1 | 253009 | -0,95627 | 0,135093 | 0,48361 | 0,356003 | 0,255282 | 0,255282 | 0,255282 | 0,846291 | 8,344622 |
| DKFZp779O | 745283 | -0,74927 | 0,502044 | -0,22155 | -0,42277 | 0,156974 | 0,156974 | 0,156974 | 0,520388 | 8,129416 |
| POLR2A | 430236 | -0,66584 | -0,23815 | -0,21254 | -0,01813 | 0,101999 | 0,101999 | 0,101999 | 0,338138 | 8,087058 |
| EEF1A2 | 2045658 | -0,70742 | 0,220364 | -0,13448 | 0,23626 | 0,133962 | 0,133962 | 0,133962 | 0,444099 | 8,024566 |
| DSC2 | 544639 | 0,627174 | -0,22197 | -0,41071 | -0,74957 | 0,06878 | 0,06878 | 0,06878 | 0,228015 | 8,003208 |
| PFKP | 950682 | -0,75493 | 0,065425 | 0,067048 | 0,100302 | 0,165906 | 0,165906 | 0,165906 | 0,549999 | 7,921909 |
| IFITM2 | 1592837 | 1,055751 | 0,630075 | 1,426945 | 2,08602 | 0,310583 | 0,310583 | 0,310583 | 1,029621 | 7,911787 |
| PREX1 | 825270 | -0,88722 | 0,410212 | -0,14103 | 0,076908 | 0,235838 | 0,235838 | 0,235838 | 0,781833 | 7,86871 |
| FKBP1A | 745496 | 0,714415 | -0,1766 | -0,06837 | 0,708429 | 0,144241 | 0,144241 | 0,144241 | 0,478176 | 7,825236 |
| CA10 | 49336 | 0,887635 | -0,61365 | -0,43032 | -0,02775 | 0,238993 | 0,238993 | 0,238993 | 0,792291 | 7,759807 |
| CASKIN2 | 46415 | -0,6818 | 0,085898 | -0,00701 | 0,06175 | 0,125828 | 0,125828 | 0,125828 | 0,417135 | 7,715386 |
| No symbol | 1591788 | 0,66976 | -0,17914 | -0,44775 | -0,43857 | 0,117718 | 0,117718 | 0,117718 | 0,39025 | 7,697548 |
| MAP1B | 629896 | 0,635084 | -0,04812 | -0,16366 | -0,37191 | 0,09658 | 0,09658 | 0,09658 | 0,320176 | 7,504428 |
| No symbol | 510608 | -1,01154 | 0,16645 | -0,31392 | 0,821248 | 0,303226 | 0,303226 | 0,303226 | 1,005232 | 7,503511 |
| DTNA | 46518 | 0,817702 | 0,052074 | 0,151802 | 1,11201 | 0,211867 | 0,211867 | 0,211867 | 0,702365 | 7,48706 |
| No symbol | 129624 | -0,81859 | -0,44512 | -0,23026 | 0,631303 | 0,21402 | 0,21402 | 0,21402 | 0,709503 | 7,42714 |
| TEAD1 | 376290 | -1,55718 | 1,533354 | -0,6021 | -2,20278 | 0,531535 | 0,531535 | 0,531535 | 1,762104 | 7,416469 |
| No symbol | 461670 | -0,665 | 0,694945 | 0,169401 | 0,714841 | 0,123965 | 0,123965 | 0,123965 | 0,410959 | 7,396788 |
| NFIX | 130046 | -0,68374 | 0,081176 | -0,18351 | -0,19485 | 0,138164 | 0,138164 | 0,138164 | 0,458032 | 7,361413 |
| AGXT2L1 | 41424 | -0,82021 | -0,05994 | -0,20041 | 0,394943 | 0,217292 | 0,217292 | 0,217292 | 0,720349 | 7,341616 |
| SERPINE2 | 246722 | 0,652486 | -0,09848 | -0,14225 | -0,5544 | 0,119269 | 0,119269 | 0,119269 | 0,395392 | 7,259798 |
| PLXNA1 | 25499 | -0,63133 | 0,743736 | -0,40471 | -1,10581 | 0,1074 | 0,1074 | 0,1074 | 0,356043 | 7,123483 |
| BIN1 | 788107 | -0,855 | 0,107667 | -0,23067 | -0,19651 | 0,241705 | 0,241705 | 0,241705 | 0,801282 | 7,108351 |
| Ells1 | 415964 | -0,80694 | 0,073656 | -0,0737 | 0,653764 | 0,218249 | 0,218249 | 0,218249 | 0,723522 | 7,073857 |
| NTRK2 | 384939 | -0,84194 | 0,041694 | -0,247 | 2,448812 | 0,237236 | 0,237236 | 0,237236 | 0,786466 | 7,039469 |
| UNC84B | 1636166 | -0,63676 | 0,268138 | 0,221742 | 0,061221 | 0,11607 | 0,11607 | 0,11607 | 0,384786 | 7,003959 |

**Supplementary table 4**.

Genes differentially expressed in patients treated with typical neuroleptics compared to controls. List ordered by decreased PenF value.

| Symbol | CLONEID | Atyp  Estimate | Typ  Estimate | NoDrug  Estimate | Uknw  Estimate | Atyp  Std Error | Typ  Std Error | NoDrug  Std Error | Uknw  Std Error | PenF |
| --- | --- | --- | --- | --- | --- | --- | --- | --- | --- | --- |
| No symbol | 378461 | -0,16327 | 1,510273 | -0,30732 | -0,16067 | 0,155048 | 0,155048 | 0,155048 | 0,514002 | 33,31869 |
| PMP22 | 133273 | 0,015055 | 1,589846 | 0,074769 | -2,41446 | 0,350424 | 0,350424 | 0,350424 | 1,161699 | 15,11594 |
| K5B | 377827 | -1,50504 | -1,13555 | 0,137698 | 3,161412 | 0,21425 | 0,21425 | 0,21425 | 0,710264 | 14,27652 |
| TM4SF1 | 840567 | 0,209659 | 1,239023 | 0,163429 | -1,14784 | 0,251769 | 0,251769 | 0,251769 | 0,834647 | 14,24021 |
| No symbol | 140574 | 0,228818 | -0,96553 | -0,40964 | 1,764552 | 0,158568 | 0,158568 | 0,158568 | 0,525671 | 13,40182 |
| MSX1 | 136188 | 0,325232 | 1,098742 | 0,035445 | -1,10797 | 0,228143 | 0,228143 | 0,228143 | 0,756324 | 12,51443 |
| No symbol | 22600 | -0,09127 | 1,006116 | -0,07238 | -1,31039 | 0,201622 | 0,201622 | 0,201622 | 0,668401 | 11,89935 |
| GNPDA1 | 207082 | 0,386967 | 1,603352 | -0,03485 | -1,85069 | 0,416814 | 0,416814 | 0,416814 | 1,38179 | 11,78415 |
| LOC91689 | 377051 | 0,088661 | 1,152785 | 0,138449 | -0,63146 | 0,263916 | 0,263916 | 0,263916 | 0,874912 | 11,65005 |
| MGC15396 | 244277 | -0,01647 | 0,896767 | 0,127921 | 0,177568 | 0,159733 | 0,159733 | 0,159733 | 0,529533 | 11,49953 |
| LHPP | 279977 | -0,83975 | 0,923486 | 0,450071 | -0,12908 | 0,181472 | 0,181472 | 0,181472 | 0,601603 | 11,02553 |
| SGK | 2013515 | -0,25079 | 0,85477 | 0,284057 | -0,49371 | 0,150908 | 0,150908 | 0,150908 | 0,500278 | 10,87393 |
| SBDSP | 77361 | -0,06072 | 0,911486 | 0,02508 | -0,8518 | 0,183259 | 0,183259 | 0,183259 | 0,607527 | 10,65111 |
| MPP1 | 296880 | 0,387371 | 1,043082 | 0,055653 | -1,41966 | 0,251351 | 0,251351 | 0,251351 | 0,833259 | 10,11216 |
| CNTNAP1 | 470279 | 0,030011 | 1,254813 | -0,11124 | -1,14239 | 0,334366 | 0,334366 | 0,334366 | 1,108466 | 10,07916 |
| PLXNA1 | 25499 | -0,63133 | 0,743736 | -0,40471 | -1,10581 | 0,1074 | 0,1074 | 0,1074 | 0,356043 | 9,885905 |
| No symbol | 261745 | -0,38743 | 0,747811 | -0,00077 | -0,47839 | 0,110694 | 0,110694 | 0,110694 | 0,366965 | 9,867822 |
| PRKACB | 362926 | 0,549367 | 0,801686 | 0,506161 | 0,319868 | 0,157191 | 0,157191 | 0,157191 | 0,521108 | 9,297376 |
| ARL6IP | 51532 | 0,238852 | 1,384483 | 0,161886 | -1,74307 | 0,406349 | 0,406349 | 0,406349 | 1,347097 | 9,14774 |
| TF | 212429 | -1,40628 | 0,833242 | -1,03522 | -0,79566 | 0,18617 | 0,18617 | 0,18617 | 0,617175 | 8,779941 |
| TOLLIP | 432656 | 0,104912 | -0,91914 | -0,58601 | 0,466061 | 0,227683 | 0,227683 | 0,227683 | 0,754796 | 8,776563 |
| APOD | 838611 | -0,3399 | 0,814343 | 0,107054 | -0,42337 | 0,178619 | 0,178619 | 0,178619 | 0,592144 | 8,688807 |
| No symbol | 137638 | 0,224125 | 0,915668 | 0,02429 | -0,87843 | 0,236077 | 0,236077 | 0,236077 | 0,782623 | 8,371904 |
| No symbol | 461670 | -0,665 | 0,694945 | 0,169401 | 0,714841 | 0,123965 | 0,123965 | 0,123965 | 0,410959 | 8,078048 |
| No symbol | 841314 | 0,22589 | 1,177912 | -0,11071 | -1,06161 | 0,359126 | 0,359126 | 0,359126 | 1,190546 | 8,002093 |
| C1QG | 292833 | 0,178459 | 0,649923 | 0,100682 | 0,976287 | 0,100843 | 0,100843 | 0,100843 | 0,334308 | 7,738043 |
| GMIP | 377672 | 0,031547 | -0,75993 | 0,206684 | 0,057228 | 0,177255 | 0,177255 | 0,177255 | 0,587623 | 7,614959 |
| SEPP1 | 530814 | 0,082835 | 1,125282 | -0,31373 | -1,17768 | 0,35288 | 0,35288 | 0,35288 | 1,169842 | 7,495205 |
| RAD51C | 26997 | -0,13824 | -0,88767 | -0,05659 | 0,569246 | 0,252343 | 0,252343 | 0,252343 | 0,836547 | 7,289529 |
| ODC1 | 796646 | -0,45414 | 1,060922 | 0,044657 | -1,48489 | 0,334063 | 0,334063 | 0,334063 | 1,10746 | 7,214359 |
| DCTN4 | 877636 | 0,188375 | 0,890691 | -0,05212 | -0,86397 | 0,256637 | 0,256637 | 0,256637 | 0,850782 | 7,193756 |
| TEAD1 | 376290 | -1,55718 | 1,533354 | -0,6021 | -2,20278 | 0,531535 | 0,531535 | 0,531535 | 1,762104 | 7,191288 |
| OLIG2 | 26884 | -0,90019 | 0,666207 | -0,25834 | -0,39787 | 0,131829 | 0,131829 | 0,131829 | 0,43703 | 7,182109 |
| RNASE1 | 840493 | -0,90456 | 0,672175 | -0,33699 | -1,57397 | 0,136144 | 0,136144 | 0,136144 | 0,451335 | 7,177064 |
| NR4A1 | 309893 | -0,37997 | -0,71272 | -0,15143 | 0,065709 | 0,163948 | 0,163948 | 0,163948 | 0,543508 | 7,124802 |
