# Supplementary material for: Inflammation-related genes up-regulated in schizophrenia brains
Source: BMC Psychiatry. 2007 Sep 6;7:46. doi: 10.1186/1471-244X-7-46 (PMC2080573; doi:10.1186/1471-244X-7-46)
Supplement: Additional file 3 — Supplementary Table 3 – Genes differentially expressed in patients treated with atypical neuroleptics compared to controls. List of 125 clones showing evidence of being differentially expressed in frontal cortex autopsy samples from schizophrenic subjects as compared to unaffected individuals. Results from experiments of hybridizing pooled mRNA samples to cDNA microarrays. [file 1471-244X-7-46-S3.doc]

## Supplementary Table 5 - Eleven oligodendrocyte and myelination related genes showed differential mRNA levels in the frontal cortex of schizophrenic subjects as compared to control individuals.

Schizophrenic subjects have been subdivided according to the antipsychotic medication received, and brain samples were hybridized onto human cDNA microarrays. The left panel illustrates with a colour code the **average** fold difference between schizophrenic subjects, treated with typical neuroleptics (T), untreated (N) or treated with atypical neuroleptics (A), compared to control individuals. Red indicates up-regulation, green down-regulation and the intensity of the colour is proportional to the magnitude of the fold change on a logarithmic scale. Clones that showed evidence of differentially expression (penalized F-ratio>7.0, *) in at least one of three schizophrenia subgroups have been listed. Only transferrin (TF) showed significant differential expression in the three groups of patients.
