# Supplementary material for: Inflammation-related genes up-regulated in schizophrenia brains
Source: BMC Psychiatry. 2007 Sep 6;7:46. doi: 10.1186/1471-244X-7-46 (PMC2080573; doi:10.1186/1471-244X-7-46)
Supplement: Additional file 4 — Supplementary Table 4 – Genes differentially expressed in patients treated with typical neuroleptics compared to controls. List of 35 clones showing evidence of being differentially expressed in frontal cortex autopsy samples from schizophrenic subjects as compared to unaffected individuals. Results from experiments of hybridizing pooled mRNA samples to cDNA microarrays. [file 1471-244X-7-46-S4.doc]

**Supplementary table 4**.

Genes differentially expressed in patients treated with typical neuroleptics compared to controls. List ordered by decreased PenF value.

| Symbol | CLONEID | Atyp  Estimate | Typ  Estimate | NoDrug  Estimate | Uknw  Estimate | Atyp  Std Error | Typ  Std Error | NoDrug  Std Error | Uknw  Std Error | PenF |
| --- | --- | --- | --- | --- | --- | --- | --- | --- | --- | --- |
| No symbol | 378461 | -0,16327 | 1,510273 | -0,30732 | -0,16067 | 0,155048 | 0,155048 | 0,155048 | 0,514002 | 33,31869 |
| PMP22 | 133273 | 0,015055 | 1,589846 | 0,074769 | -2,41446 | 0,350424 | 0,350424 | 0,350424 | 1,161699 | 15,11594 |
| K5B | 377827 | -1,50504 | -1,13555 | 0,137698 | 3,161412 | 0,21425 | 0,21425 | 0,21425 | 0,710264 | 14,27652 |
| TM4SF1 | 840567 | 0,209659 | 1,239023 | 0,163429 | -1,14784 | 0,251769 | 0,251769 | 0,251769 | 0,834647 | 14,24021 |
| No symbol | 140574 | 0,228818 | -0,96553 | -0,40964 | 1,764552 | 0,158568 | 0,158568 | 0,158568 | 0,525671 | 13,40182 |
| MSX1 | 136188 | 0,325232 | 1,098742 | 0,035445 | -1,10797 | 0,228143 | 0,228143 | 0,228143 | 0,756324 | 12,51443 |
| No symbol | 22600 | -0,09127 | 1,006116 | -0,07238 | -1,31039 | 0,201622 | 0,201622 | 0,201622 | 0,668401 | 11,89935 |
| GNPDA1 | 207082 | 0,386967 | 1,603352 | -0,03485 | -1,85069 | 0,416814 | 0,416814 | 0,416814 | 1,38179 | 11,78415 |
| LOC91689 | 377051 | 0,088661 | 1,152785 | 0,138449 | -0,63146 | 0,263916 | 0,263916 | 0,263916 | 0,874912 | 11,65005 |
| MGC15396 | 244277 | -0,01647 | 0,896767 | 0,127921 | 0,177568 | 0,159733 | 0,159733 | 0,159733 | 0,529533 | 11,49953 |
| LHPP | 279977 | -0,83975 | 0,923486 | 0,450071 | -0,12908 | 0,181472 | 0,181472 | 0,181472 | 0,601603 | 11,02553 |
| SGK | 2013515 | -0,25079 | 0,85477 | 0,284057 | -0,49371 | 0,150908 | 0,150908 | 0,150908 | 0,500278 | 10,87393 |
| SBDSP | 77361 | -0,06072 | 0,911486 | 0,02508 | -0,8518 | 0,183259 | 0,183259 | 0,183259 | 0,607527 | 10,65111 |
| MPP1 | 296880 | 0,387371 | 1,043082 | 0,055653 | -1,41966 | 0,251351 | 0,251351 | 0,251351 | 0,833259 | 10,11216 |
| CNTNAP1 | 470279 | 0,030011 | 1,254813 | -0,11124 | -1,14239 | 0,334366 | 0,334366 | 0,334366 | 1,108466 | 10,07916 |
| PLXNA1 | 25499 | -0,63133 | 0,743736 | -0,40471 | -1,10581 | 0,1074 | 0,1074 | 0,1074 | 0,356043 | 9,885905 |
| No symbol | 261745 | -0,38743 | 0,747811 | -0,00077 | -0,47839 | 0,110694 | 0,110694 | 0,110694 | 0,366965 | 9,867822 |
| PRKACB | 362926 | 0,549367 | 0,801686 | 0,506161 | 0,319868 | 0,157191 | 0,157191 | 0,157191 | 0,521108 | 9,297376 |
| ARL6IP | 51532 | 0,238852 | 1,384483 | 0,161886 | -1,74307 | 0,406349 | 0,406349 | 0,406349 | 1,347097 | 9,14774 |
| TF | 212429 | -1,40628 | 0,833242 | -1,03522 | -0,79566 | 0,18617 | 0,18617 | 0,18617 | 0,617175 | 8,779941 |
| TOLLIP | 432656 | 0,104912 | -0,91914 | -0,58601 | 0,466061 | 0,227683 | 0,227683 | 0,227683 | 0,754796 | 8,776563 |
| APOD | 838611 | -0,3399 | 0,814343 | 0,107054 | -0,42337 | 0,178619 | 0,178619 | 0,178619 | 0,592144 | 8,688807 |
| No symbol | 137638 | 0,224125 | 0,915668 | 0,02429 | -0,87843 | 0,236077 | 0,236077 | 0,236077 | 0,782623 | 8,371904 |
| No symbol | 461670 | -0,665 | 0,694945 | 0,169401 | 0,714841 | 0,123965 | 0,123965 | 0,123965 | 0,410959 | 8,078048 |
| No symbol | 841314 | 0,22589 | 1,177912 | -0,11071 | -1,06161 | 0,359126 | 0,359126 | 0,359126 | 1,190546 | 8,002093 |
| C1QG | 292833 | 0,178459 | 0,649923 | 0,100682 | 0,976287 | 0,100843 | 0,100843 | 0,100843 | 0,334308 | 7,738043 |
| GMIP | 377672 | 0,031547 | -0,75993 | 0,206684 | 0,057228 | 0,177255 | 0,177255 | 0,177255 | 0,587623 | 7,614959 |
| SEPP1 | 530814 | 0,082835 | 1,125282 | -0,31373 | -1,17768 | 0,35288 | 0,35288 | 0,35288 | 1,169842 | 7,495205 |
| RAD51C | 26997 | -0,13824 | -0,88767 | -0,05659 | 0,569246 | 0,252343 | 0,252343 | 0,252343 | 0,836547 | 7,289529 |
| ODC1 | 796646 | -0,45414 | 1,060922 | 0,044657 | -1,48489 | 0,334063 | 0,334063 | 0,334063 | 1,10746 | 7,214359 |
| DCTN4 | 877636 | 0,188375 | 0,890691 | -0,05212 | -0,86397 | 0,256637 | 0,256637 | 0,256637 | 0,850782 | 7,193756 |
| TEAD1 | 376290 | -1,55718 | 1,533354 | -0,6021 | -2,20278 | 0,531535 | 0,531535 | 0,531535 | 1,762104 | 7,191288 |
| OLIG2 | 26884 | -0,90019 | 0,666207 | -0,25834 | -0,39787 | 0,131829 | 0,131829 | 0,131829 | 0,43703 | 7,182109 |
| RNASE1 | 840493 | -0,90456 | 0,672175 | -0,33699 | -1,57397 | 0,136144 | 0,136144 | 0,136144 | 0,451335 | 7,177064 |
| NR4A1 | 309893 | -0,37997 | -0,71272 | -0,15143 | 0,065709 | 0,163948 | 0,163948 | 0,163948 | 0,543508 | 7,124802 |
